# *In silico* identification and biophysical characterization of candidate antimicrobial peptides from the Indian marine microbiome targeting multidrug-resistant ESKAPE pathogens

**DOI:** 10.1371/journal.pone.0353985

**Published:** 2026-07-22

**Authors:** Sreelakshmi K. V, Nasri Thaha, Budheswar Dehury

**Affiliations:** Department of Bioinformatics, Manipal School of Life Sciences, Manipal Academy of Higher Education, Manipal, Karnataka, India; Albert Einstein College of Medicine, UNITED STATES OF AMERICA

## Abstract

The global health crisis of antimicrobial resistance necessitates the discovery of new antibacterial agents. Underexplored marine microbiomes, particularly from the biodiverse Indian coast, represent a rich potential source of antimicrobial peptides (AMPs). Targeting the urgent threat of multidrug-resistant ESKAPE pathogens, the present study aimed to computationally identify novel, membrane-active AMPs from these unique metagenomic datasets, with a focus on inhibiting Gram-negative bacteria. In this study, we computationally mined Indian marine high-resolution shotgun metagenomic datasets through quality filtering, de novo assembly, and small open reading frame prediction. An ensemble of six machine learning-based AMP prediction tools identified over 51,000 high-confidence candidate AMPs. Subsequent filtering based on physicochemical properties and AlphaFold3-predicted structures prioritized ten peptides with favourable membrane-active characteristics. Two lead candidates, c_AMP_1 and c_AMP_2, were subjected to all-atom molecular dynamics simulations within Gram-negative membrane mimetic models of *Pseudomonas aeruginosa*, *Acinetobacter baumannii*, and *Klebsiella pneumoniae*. Our simulations indicated distinct membrane interaction modes: c_AMP_1 adopted a stable, surface-associated α-helical orientation, while c_AMP_2 displayed a more flexible, membrane-inserting orientation in the simulations. Analysis of the MD simulations revealed distinct predicted peptide-membrane interaction profiles, characterized by specific hydrogen bonding patterns, peptide tilt angles, and membrane thinning, which collectively suggest differing biophysical interaction modes. Taken together, our work suggests the Indian marine microbiome as a promising reservoir for novel AMP candidates and suggests that an integrated computational pipeline – combining machine learning, structural biology, and biophysical simulation – may help prioritize candidate peptides for future experimental validation against critical pathogens.

## 1. Introduction

The rise of antimicrobial resistance (AMR) has become a critical global health concern in the modern era. The proliferation of resistant bacterial strains increasingly undermines the efficacy of conventional antibiotics, which threatens to reverse decades of medical progress in treating infectious diseases [1]. Recent comprehensive analyses estimated that bacterial AMR was associated with approximately 4.71 million deaths worldwide in 2021, with 1.14 million deaths directly attributable to resistant infections, underscoring the gravity of this crisis [2]. The primary drivers of AMR include the overuse and misuse of antibiotics in human and veterinary medicine, inadequate infection prevention and control measures, and the environmental dissemination of resistance genes facilitated by anthropogenic activities [3]. The uneven distribution of the AMR burden complicates this issue significantly, especially for low- and middle-income countries (LMICs). In these regions, the lack of access to diagnostic services and second-line therapies exacerbates health problems, resulting in increased morbidity and mortality [4]. India, in particular, bears one of the highest burdens of drug-resistant infections globally and reported nearly 300,000 deaths attributable to AMR in 2019 alone [5]. Due to the growing challenge of AMR, organizations such as the World Health Organization (WHO) have emphasized the need for comprehensive global action, including the development of new antimicrobial therapies, enhanced surveillance, and coordinated international efforts to limit its progression.

Within the spectrum of AMR-related challenges, ESKAPE pathogens, comprising *Enterococcus faecium*, *Staphylococcus aureus*, *Klebsiella pneumoniae*, *Acinetobacter baumannii*, *Pseudomonas aeruginosa*, and *Enterobacter species*, have been highlighted because of their prominent role in nosocomial infections and notorious ability to “escape” the lethal action of antibiotics. These pathogens account for a significant fraction of hospital-acquired infections worldwide and exhibit multidrug resistance patterns that severely limit therapeutic options [6]. Studies conducted in diverse healthcare settings, including tertiary hospitals across South Asia and the Middle East, have revealed an alarmingly high prevalence of multidrug-resistant (MDR) strains within the ESKAPE group, with notable resistance to critical antibiotics such as carbapenems and oxacillin [7]. In India, the increasing threat posed by ESKAPE pathogens is especially concerning. A study that examined 20,177 isolates revealed that *Acinetobacter baumannii* was the most prevalent, accounting for 35.9% of the total. It was followed by *Pseudomonas aeruginosa* at 25.3% and *Klebsiella pneumoniae* at 19.5% [8]. The clinical and economic burden of infections caused by these pathogens is substantial, necessitating rigorous infection control measures, antimicrobial stewardship programs, and accelerated development of novel therapeutic agents, as articulated in recent WHO priority pathogen lists. The persistence and adaptability of ESKAPE bacteria highlight an urgent need for innovative strategies that transcend traditional antibiotic development paradigms.

Antimicrobial peptides (AMPs) have emerged as promising candidates in the battle against multidrug-resistant pathogens, including the ESKAPE group. These short, naturally occurring peptides demonstrate strong and broad-spectrum antimicrobial activity, with the ability to target a wide range of microorganisms, including bacteria, viruses, fungi, and even cancerous cells [9,10]. Their primary mode of action involves interacting with microbial membranes through electrostatic attractions, followed by insertion into the membrane due to their amphipathic nature, ultimately leading to membrane disruption and cell lysis. In addition to their membrane-targeting effects, some AMPs can penetrate microbial cells and interfere with essential intracellular processes such as nucleic acid synthesis and protein folding [11]. Unlike conventional antibiotics, which typically target singular bacterial processes, AMPs engage multiple pathways concurrently, rendering the evolution of resistance more difficult for pathogens. Their rapid bactericidal action coupled with their immunomodulatory properties, such as the recruitment of immune cells and the regulation of inflammatory responses, confers additional therapeutic advantages [12]. Furthermore, AMPs display preferential selectivity towards bacterial membranes over mammalian cells due to differences in membrane composition, which translates to relatively low cytotoxicity [13]. Recent advancements have also demonstrated that AMPs can synergize with existing antibiotics, restoring susceptibility in resistant strains, as exemplified by LL-37’s ability to resensitize colistin-resistant bacteria [14]. Collectively, these attributes position AMPs as promising candidates for next-generation antimicrobials, with potential applications spanning systemic infections, wound healing, and biofilm disruption.

Marine ecosystems have garnered significant attention as reservoirs of untapped AMP diversity, driven by their unique environmental conditions that foster the evolution of microorganisms with novel bioactive compounds [15,16]. The marine biome encompasses a vast array of habitats characterized by high salinity, variable pressure, and nutrient limitations, selecting for microbes equipped with specialized metabolic pathways and distinctive antimicrobial properties [17,18]. Recent large-scale metagenomic studies have expanded our understanding of global marine microbial diversity, revealing an unprecedented number of biosynthetic gene clusters (BGCs) responsible for the synthesis of novel bioactive molecules, including AMPs, many of which show low sequence similarity to previously identified peptides, suggesting the existence of entirely new antimicrobial scaffolds [19,20]. This emphasizes the vast potential of marine environments as a valuable source for identifying novel and potent antimicrobial peptides.

The Indian marine microbiome harbors exceptional taxonomic and functional diversity yet remains significantly underexplored as a source for AMP discovery and bioprospecting. India’s extensive coastline, spanning over 7,500 kilometres and encompassing diverse ecosystems such as mangroves, coral reefs, and deep-sea sediments, offers a unique milieu for microbial innovation [21,22]. Metagenomic surveys across various Indian aquatic systems have revealed high bacterial diversity, including genera known for antibiotic production and resistance gene reservoirs [18,23]. However, the majority of these ecosystems remain inadequately characterized in terms of their AMP repertoire, offering immense scope for novel peptide discovery.

The integration of artificial intelligence (AI) and machine learning (ML) techniques into AMP discovery pipelines has significantly enhanced the efficiency of screening, prediction, and optimization processes across large-scale metagenomic datasets [24,25]. Traditional wet-lab approaches, while essential, are constrained by time, cost, and scale, limiting the pace of novel AMP identification. By leveraging advanced pattern recognition, sequence-based feature extraction, and predictive modeling, AI/ML approaches enable the identification of antimicrobial potential, toxicity profiles, and possible mechanisms of action directly from primary peptide sequences, substantially accelerating early-stage discovery and development efforts [26,27]. Recent advances in deep learning, using architectures such as convolutional and recurrent neural networks, graph-based models, and protein language models adapted from natural language processing, have significantly improved the accuracy of AMP classification and functional annotation. These models can capture subtle structural and physicochemical characteristics, enhancing the prediction of peptide efficacy [28,29]. The integration of high-throughput metagenomic sequencing with advanced machine learning algorithms has further accelerated the pace of AMP discovery. A landmark study by Santos-Júnior et al. exemplified this synergy, identifying nearly one million AMP candidates from global metagenomes, including those from marine ecosystems, over 90% of which were novel and previously uncharacterized [30]. The integration of AI/ML with well-curated AMP databases enables a data-driven and scalable framework for mining Indian marine metagenomes, making the discovery process more systematic, efficient, and rapid.

The present study aims to computationally prioritize and biophysically characterize candidate AMPs from Indian marine metagenomic datasets with predicted activity against ESKAPE pathogens. By integrating advanced machine learning-based AMP prediction tools, structural modelling, and all-atom molecular dynamics simulations, this study identifies and characterizes candidate membrane-active peptides from metagenomic data. Crucially, all-atom molecular dynamics simulations were performed in biologically relevant membrane models – an asymmetric outer membrane model for *Acinetobacter baumannii* and symmetric inner membrane models for *Klebsiella pneumoniae* and *Pseudomonas aeruginosa* – enabling detailed insights into predicted peptide-membrane interaction modes, orientation states, and conformational dynamics. This integrated computational framework aids in the discovery and prioritization of candidate AMPs and provides biophysical characterization of their predicted membrane interaction profiles, providing a computational basis for future experimental validation. Our findings contribute to a deeper understanding of how marine-derived AMPs may overcome bacterial resistance mechanisms through membrane targeting. This study not only highlights the untapped potential of India’s marine microbial diversity in drug discovery but also supports broader national and global initiatives such as the blue economy, “Make in India,” and the One Health framework. Ultimately, sustainable development goals (SDGs) 3 and 14 are advanced by the promotion of novel antimicrobial strategies and sustainable marine resource utilization.

## 2. Materials and methods

### 2.1. Dataset selection and acquisition

Five publicly available whole-genome shotgun (WGS) metagenomic datasets were retrieved from the National Center for Biotechnology Information (NCBI) BioProjects and the European Nucleotide Archive (ENA). Dataset selection was performed using targeted search queries containing marine-related keywords in conjunction with the term “India,” with additional filtering for experiments explicitly designated as “METAGENOMIC” in their library source metadata. Stringent inclusion criteria were applied to ensure dataset relevance and quality: (i) data type specified as WGS metagenomic sequences, (ii) submission date after January 1, 2020, (iii) sequencing platform restricted to Illumina to maintain consistency in read quality and format, and (iv) minimum project size of 5 samples to ensure robust representation.

### 2.2. Sequence pre-processing, assembly, and gene prediction

Raw sequencing reads in FASTQ format were downloaded for all selected metagenomic samples. The initial quality assessment of sequencing data was performed using FastQC to examine base quality, adapter contamination, and other key quality parameters. The reads were then processed using Trimmomatic [31], which removes low-quality bases and adapter sequences based on stringent filtering parameters (quality score threshold: Q ≥ 25; minimum read length: 60 bp). After filtering, FastQC was rerun to confirm improvements in quality. High-quality, adapter-free reads were subsequently assembled de novo into contigs using MEGAHIT (v1.1) [32], which was optimized for large and complex metagenomic datasets. Assembly was performed using the parameters --min-count 2 --k-min 33 --k-max 63 --k-step 10 for each sample, generating contigs that served as the basis for downstream gene prediction and peptide mining. Small open reading frames (smORFs) ranging from 33–303 base pairs were predicted from the assembled contigs using MetaProdigal [33], enabling the identification of potential coding sequences from metagenomic assemblies. To minimize redundancy yet preserve structural and functional diversity, the protein sequences translated from these smORFs were clustered using CD-HIT [34]. Clustering was performed with the following parameters: -n 2 -p 1 -c 0.5 -d 200 -M 50000 -l 5 -s 0.95 -aL 0.95 -g 1, requiring family members to share atleast 50% sequence homology [16]. Additionally, shorter sequences were required to be at least 95% the length of the cluster representative, and alignments were required to cover at least 95% of the longer sequence. The resulting non-redundant set of smORF-encoded proteins was retained for subsequent AMP prediction.

### 2.3. ML-based AMP prediction and novelty assessment

Six distinct machine learning-based tools were used to assess the antimicrobial potential of the non-redundant peptide dataset. These included AMPlify [35], ampir [36], AMPScanner v2 [29], AI4AMP [37], amPEPpy [38], and APIN [39]. Each tool applies distinct algorithmic strategies and sequence features for AMP classification, including physicochemical descriptors, evolutionary information, and deep learning embeddings. Only peptides that were consistently predicted as AMPs by all six tools were retained for further evaluation, suggesting a high-confidence set of candidate antimicrobial peptides. The confidence of this consensus-based approach is supported by the independent benchmarking of each constituent tool against curated AMP and non-AMP reference datasets in their respective original publications, with reported accuracies ranging from 85% to 97%. However, no additional independent benchmarking was performed within this study, which represents a limitation of the current pipeline. To assess the novelty of the predicted AMPs, BLASTp searches were conducted against CAMP R4 and other curated AMP databases using an E-value cut-off of 10 ⁻ ⁵ and a maximum sequence identity threshold of 40%. Peptides showing low similarity to known AMPs were retained as putative novel candidates.

### 2.4. Cell-penetrating peptide (CPP) screening and physicochemical profiling

High-confidence AMPs were further evaluated for their cell-penetrating potential using two complementary machine learning-based CPP prediction tools: pLM4CPPs [40] and CellPPD-Mod [41]. Peptides shorter than 50 amino acids and containing both arginine (R) and tryptophan (W) residues were prioritized due to their established roles in mediating membrane interaction and cellular uptake. For CellPPD-Mod, peptides with a prediction score greater than 0 were classified as CPPs (default threshold). For pLM4CPPs, the default classification threshold was applied. Additionally, similarity searches were performed against CPPsite 3.0 [42], an updated curated database of 4143 experimentally validated CPPs, using the Smith-Waterman algorithm, to assess the sequence similarity of shortlisted candidates to known CPPs. The top 10 candidates were subjected to physicochemical property analysis via the DBAASP server [43], with parameters calculated on the Moon-Fleming hydrophobicity scale. Parameters such as the net charge, hydrophobicity, and isoelectric point were computed to assess their suitability for further evaluation. *In silico* toxicity and haemolytic activity of all ten shortlisted peptides were additionally assessed using ToxinPred 3.0 [44] and HemoPI 2.0 [45] respectively, to obtain a preliminary predicted safety profile. On the basis of these properties, only two peptides with optimal antimicrobial potential and desirable biochemical profiles were advanced to structural modelling and all-atom molecular dynamics simulations. The selection was guided by a multi-criteria evaluation framework that considered the highest consensus scores across all six machine learning prediction tools, contrasting yet complementary physicochemical profiles, and structurally distinct AlphaFold3-predicted conformations enabling a meaningful comparative biophysical analysis. The considerable computational demands of 300 ns all-atom simulations across three bacterial membrane systems further necessitated a focused selection of the most structurally divergent candidates. Three-dimensional structural models of the selected peptides were generated using AlphaFold3 [46], enabling accurate structural representation for interaction studies.

### 2.5. System preparation for molecular dynamics simulations

Biologically relevant membrane models have been developed to simulate the interaction between antimicrobial peptides and bacterial membranes. For this study, we focused on three clinically significant Gram-negative bacteria: *Klebsiella pneumoniae*, *Pseudomonas aeruginosa*, and *Acinetobacter baumannii*. Symmetric inner membrane models for K. pneumoniae and P. aeruginosa were constructed based on aggregated lipidomic data, incorporating detailed information on both lipid headgroup and tail compositions to closely mimic native bacterial membranes [47]. In contrast, an asymmetric outer membrane model was specifically designed for A. baumannii, reflecting the distinct lipid distributions in its outer and inner leaflets, as reported in species-specific lipidomic studies [48]. All membrane systems were generated using the CHARMM-GUI Membrane Builder, a widely used tool for creating all-atom lipid bilayer structures. The CHARMM36 force field was utilized to precisely assign lipid topologies and parameterize interatomic interactions with high fidelity [49]. Each membrane bilayer was placed within a tetragonal simulation box of sufficient size to include peptides, water molecules, and ions, thereby maintaining a realistic aqueous environment. It should be noted that the three membrane models employed are not architecturally equivalent. The *A. baumannii* model incorporates an asymmetric outer membrane containing Lipid A/LPS, reflecting its biologically distinct outer membrane composition, while the *K. pneumoniae* and *P. aeruginosa* models represent symmetric inner membrane phospholipid bilayers lacking LPS, based on available validated lipidomic data. This distinction was necessitated by the availability of species-specific validated membrane compositions and reflects the current state of bacterial membrane modeling resources. Accordingly, cross-species comparisons of peptide-membrane interaction metrics should be interpreted with this architectural difference in mind. Initial peptide placement and orientation relative to the membrane were handled using the default parameters of CHARMM-GUI Membrane Builder, which automatically positions the peptide in the aqueous phase above the membrane surface during system assembly. The two selected peptides, c_AMP_1 and c_AMP_2, were each simulated independently in all three bacterial membrane models (*A. baumannii*, *K. pneumoniae*, and *P. aeruginosa*), yielding a total of six independent MD simulation systems (two peptides × three membranes). No additional peptides were carried forward to the MD stage. The detailed lipid compositions used for each membrane system are summarized in Table 1.

**Table 1:** Lipid composition of bacterial membrane systems used in MD simulations. The table summarizes the distributions of major phospholipid species—phosphatidylethanolamine (PE), phosphatidylglycerol (PG), and cardiolipin (CL)—in the upper and lower leaflets of the simulated membranes for *Acinetobacter baumannii*, *Klebsiella pneumoniae*, and *Pseudomonas aeruginosa.*

Bacterium	Leaflet	Phosphatidylethanolamine (PE)	Phosphatidylglycerol (PG)	Cardiolipin (CL)	Lipid A/LPS
***A. baumannii* (OM)**	Upper (outer)	18%	4%	3%	75% hepta-acyl lipid A
	Lower (inner)	72%	16%	12%	–
***K. pneumoniae***	Symmetric	80%	15%	5%	–
***P. aeruginosa***	Symmetric	65%	23%	12%	–

### 2.6. All-Atom MD simulations

GROMACS version 2023.1 was used to perform all-atom molecular dynamics (MD) simulations using deprotonated lipids and the CHARMM36 force field [50,51]. TIP3P water molecules were introduced into the structure created by CHARMM-GUI to completely submerge the system and replicate an aqueous environment that is biologically relevant. Counter-ions were introduced using a Monte Carlo algorithm to neutralize the system’s net charge, and sodium chloride (NaCl) was included at physiological concentrations to mimic the ionic milieu of bacterial cells [52,53]. Energy minimization was achieved using the steepest descent method, followed by a 1 ns equilibration period in which the temperature was gradually increased from 100 K to 310.15 K. A 300 ns production run was then conducted under constant pressure and temperature (NPT) conditions, maintaining 310.15 K and 1 atm. Each simulation system was run as a single trajectory. While multiple replicates would further strengthen statistical confidence, the computational demands of six independent 300 ns all-atom simulations precluded this in the current study. Long-range electrostatics were handled with the particle-mesh Ewald (PME) method, and a 1 nm cut-off was applied for short-range interactions.

### 2.7. Post-MD analysis

All molecular dynamics simulation analyses were conducted using GROMACS [50] and VMD [54], with PyMOL employed for structural visualization. Structural stability and flexibility were evaluated through the root mean square deviation (RMSD) and root mean square fluctuation (RMSF), whereas the compactness of each peptide was assessed using the radius of gyration. Solvent accessibility was analysed via solvent-accessible surface area (SASA) calculations. Peptide-membrane interactions were assessed by quantifying hydrogen bonds, defined using a donor-acceptor distance cutoff of ≤3.0 Å and a donor-hydrogen-acceptor angle of ≥150°, consistent with standard GROMACS hydrogen bond criteria and by calculating the membrane tilt angle to evaluate peptide orientation relative to the membrane normal. The membrane thickness and area per lipid were further quantified using FATSLiM [55]. The evolution of secondary structural elements over the simulation trajectory was examined using DSSP, and contact maps were generated using CONAN to identify persistent residue-level interactions within the peptide [56]. Additionally, the C.O.M. of in-contact peptide residues was monitored to track peptide positioning and orientation during membrane binding. Representative system snapshots were extracted every 100 ns to visualize the dynamic conformational changes throughout the 300 ns simulations.

## 3. Results

### 3.1. Identification and characterization of candidate AMPs from Indian marine microbiomes

To explore the antimicrobial potential of Indian marine microbiomes, we selected five high-quality metagenomic datasets deposited in the NCBI BioProject, which represent diverse ecological niches such as seawater, sponge, sediment, and coral environments across the Indian coastline. The datasets - PRJNA822508, PRJNA891635, PRJNA900060, PRJNA928230, and PRJNA971765 – comprised a total of 59 metagenomic samples, all sequenced using Illumina platforms after 2020 (**Table A in** **S1 File**). Overall, they yielded over 925 million raw sequencing reads. After quality control using Trimmomatic (Q ≥ 25, minimum read length ≥ 60 bp), approximately 85.8% of the reads were retained as high-quality, adapter-free sequences. These trimmed reads were assembled using MEGAHIT with a multi-kmer strategy, generating ~32.6 million contigs across all datasets. From these assemblies, ~ 35.2 million open reading frames (ORFs) were predicted using MetaProdigal, of which ~29.1 million (82.6%) were classified as small ORFs (smORFs) in the range of 33–303 bp (**Table B in** **S1 File**).

To remove redundancy and enrich for structurally unique peptides, we applied CD-HIT clustering with stringent identity thresholds (≥50% identity, ≥ 95% coverage). This reduced the dataset to ~24.2 million non-redundant smORFs, representing approximately 83.1% of the original smORF space. These peptides were then screened using six state-of-the-art machine learning-based AMP prediction tools: AMPScanner v2, AMPlify, APIN, ampir, AI4AMP, and amPEPpy. Only sequences that were consistently classified as AMPs by all six tools were retained, resulting in a total of 51,185 c_AMPs, representing ~0.21% of the non-redundant dataset (**S2 File**).

The c_AMPs were further filtered using a length cut-off of ≤50 amino acids and required the presence of both arginine (R) and tryptophan (W) residues, with potential membrane-penetrating ability. The peptides were subsequently evaluated using pLM4CPPs and CellPPD-Mod to identify potential cell-penetrating candidates. Nine of the ten shortlisted peptides were consistently predicted as CPPs by both tools, supporting their cell-penetrating potential. One peptide, c_AMP_6, yielded conflicting predictions, classified as CPP by pLM4CPPs but as Non-CPP by CellPPD-Mod, and its cell-penetrating ability is noted as requiring experimental confirmation. CPP prediction results are summarized in **Table C in** **S1 File**. Similarity searches against CPPsite 3.0 revealed low alignment scores [13–19] across short local windows (3–9 residues) for all ten candidates, and the results are provided in **Table D in** **S1 File**. The final set of 10 peptides underwent physicochemical characterization using the DBAASP server, which indicated that they were strongly cationic (net charges between +4 and +11), had high isoelectric points, and displayed moderate to high levels of hydrophobicity and amphipathic properties (Table 2).

**Table 2:** Sequences and physicochemical features of the top 10 predicted membrane-penetrating antimicrobial peptides (c_AMPs). The physicochemical properties of the antimicrobial peptides, including sequence length, net charge, hydrophobicity etc., were calculated.

ID	Sequence	Length	Normalized Hydrophobic Moment	Normalized Hydrophobicity	Net Charge	Isoelectric Point	Penetration Depth	Amphiphilicity Index
**c_AMP_1**	RWVAKRTRKFPRKYTQVAKKKTLLARLILYLIG	33	1.2	0.38	11	12.02	26	1.59
**c_AMP_2**	GHDEAKAFMTCGLAGKRGGKAPRRWQHLGNMLNRLLSCRS	40	0.57	0.69	6	11.26	21	0.89
**c_AMP_3**	FWLLGRWLRGLWRKRKAEQAAS	22	0.62	0.62	5	12.13	18	1.84
**c_AMP_4**	DLGIRTAEKLEKKIRWFIKGRKAVKKLFEKEARHLNCF	38	1.17	0.7	7	10.85	30	1.38
**c_AMP_5**	RWVSKRIRKFPRKYKHILRKTILYFIGS	28	1.3	0.63	10	12.01	15	1.75
**c_AMP_6**	GSSFLKGGLCGRKSGGLLQVLQRWIKG	27	0.74	0.03	5	11.49	14	0.94
**c_AMP_7**	KKAARDHKRWWQVARHTARLIVGSAA	26	1.12	0.74	6	12.14	16	1.49
**c_AMP_8**	REYGKRLRTGNAPLLLTGGVLALAGLLGGKRGWRRWARLALIVAPLLRRR	50	0.49	0.03	11	12.48	14	1.04
**c_AMP_9**	LSRPPDVGMRWKWVLAAAAAKAALCGWHTTLLQAKTKAATLV	42	0.13	−0.3	5	11.07	2	1.03
**c_AMP_10**	PRWMRRFNRGMALLLLLSAWAAAFW	25	0.59	−0.42	4	12.58	12	1.22

*In silico* safety profiling revealed that all ten shortlisted peptides were predicted as non-toxic by ToxinPred 3.0. HemoPI 2.0 predicted nine out of ten peptides as hemolytic based on binary classification; however, predicted HC50 values ranged from 18.3 to 126.4 μM, indicating variable but moderate predicted hemolytic potency. Notably, c_AMP_2, one of the two lead candidates selected for MD simulations, exhibited a higher HC50 value of 65.6 μM, and c_AMP_7 was classified as non-hemolytic with an HC50 of 126.4 μM, suggesting comparatively lower predicted hemolytic activity among the shortlisted candidates. These *in silico* predictions provide a preliminary safety assessment and should be confirmed through experimental hemolysis assays. The complete safety prediction results are summarized in **Table G in** **S1 File**.

### 3.2. Structural and functional characterization of selected peptides

Predicted structures of the ten shortlisted cell-penetrating antimicrobial peptides and their corresponding helical wheel diagrams revealed conserved amphipathic features and structural diversity among candidates (**Fig. A in** **S1 File** and **Fig. B in** **S1 File**). Among these, c_AMP_1 and c_AMP_2 were the only two peptides advanced to all-atom MD simulations, selected based on their contrasting physicochemical profiles, specifically net charge (+11 vs +6), hydrophobic moment, and amphiphilicity index, structurally distinct AlphaFold3-predicted conformations, and highest consensus scores across all six ML prediction tools, enabling a meaningful comparative biophysical analysis. c_AMP_1 demonstrated a high net positive charge of +11, an amphiphilicity index of 1.59, and a membrane penetration depth of 26 Å, suggesting strong electrostatic interactions and a predicted potential for membrane insertion. In comparison, c_AMP_2 exhibited a lower net charge (+6), moderate amphiphilicity (0.89), and a shallower insertion depth of 21 Å, reflecting a more balanced distribution of hydrophobic and polar residues.

**Fig 1:**
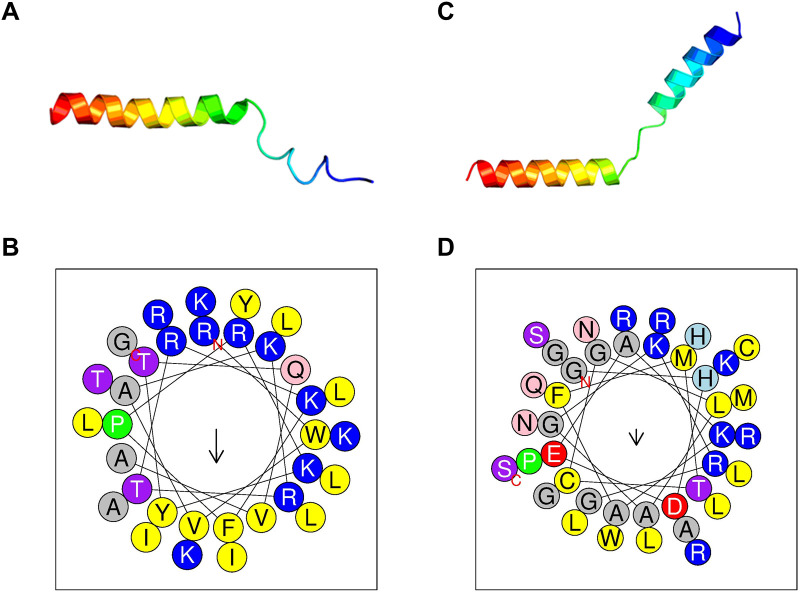
Predicted structural features of c_AMP_1 and c_AMP_2. (A) Predicted 3D structure of c_AMP_1 showing a distinct α-helical conformation. (B) A helical wheel representation of c_AMP_1 depicts the arrangement of amino acids, with blue highlighting cationic residues and yellow representing hydrophobic side chains. (C) Predicted 3D structure of c_AMP_2 highlighting α-helical regions. (D) The helical wheel diagram of c_AMP_2 visualizes residue distribution, where blue corresponds to positively charged amino acids and yellow indicates hydrophobic regions.

AlphaFold3-based structural predictions showed that c_AMP_1 adopts a continuous α-helical conformation (Fig 1A), enriched with basic residues like Arg and Lys. Its helical wheel diagram confirmed a classic amphipathic layout, segregating hydrophobic and polar residues (Fig 1B). In contrast, c_AMP_2 forms a semi-helical structure with flexible termini (Fig 1C), while retaining a clearly amphipathic core region (Fig 1D). These structural features are consistent with typical membrane-active AMPs, predicted to orient favorably in bacterial bilayers. Together, these parameters supported the selection of c_AMP_1 and c_AMP_2 as representative peptides for further structural and dynamic evaluation in lipid bilayer systems.

### 3.3. Structural stability and conformational flexibility

**Fig 2:**
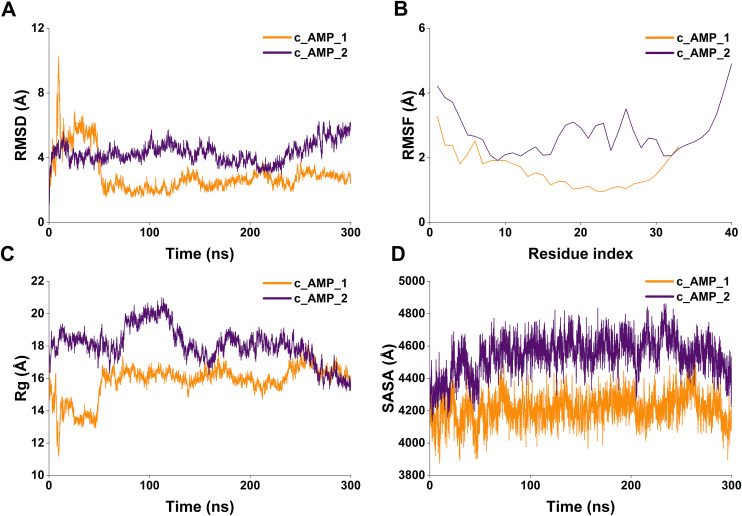
Structural dynamics of c_AMP_1 and c_AMP_2 in the *A. baumannii* membrane. (A) RMSD trajectories showing backbone stability over time. (B) RMSF plots reflecting residue-level flexibility. (C) SASA data illustrating how much of the peptide remained in contact with the surrounding solvent. (D) Rg profiles highlight the structural compactness of the peptides during simulations.

The structural stability and flexibility of c_AMP_1 and c_AMP_2 were evaluated in the outer membrane of *A. baumannii* and the symmetric membranes of *K. pneumoniae* and *P. aeruginosa* over 300 ns of all-atom MD simulations. Metrics including RMSD, RMSF, SASA, and Rg were used to assess conformational dynamics and membrane engagement. In the *A. baumannii* outer membrane, c_AMP_1 achieved equilibrium within the first 40−50 ns, maintaining stable RMSD values ranging between 1.25 and 1.35 Å (Fig 2A). A minor transient increase (~1.5 Å) occurred near 120 ns but quickly returned to baseline. The RMSF profile showed minimal fluctuations within the central helical region (residues 12−28), while terminal residues exhibited higher mobility, with a peak of 2.3 Å at the C-terminus (Fig 2B). c_AMP_2 exhibited more flexibility, stabilizing around 2.3–2.6 Å after 80 ns, with RMSF values reaching ~3.0 Å at the C-terminal end. SASA analysis revealed that c_AMP_1 was more solvent-exposed (~49.6 nm²), suggesting surface association, while c_AMP_2 maintained a lower SASA (~31.4 nm²), consistent with deeper membrane embedding (Fig 2C). Rg values remained stable, with c_AMP_1 averaging ~1.36 nm and c_AMP_2 showing tighter compaction at ~1.14 nm (Fig 2D).

**Fig 3:**
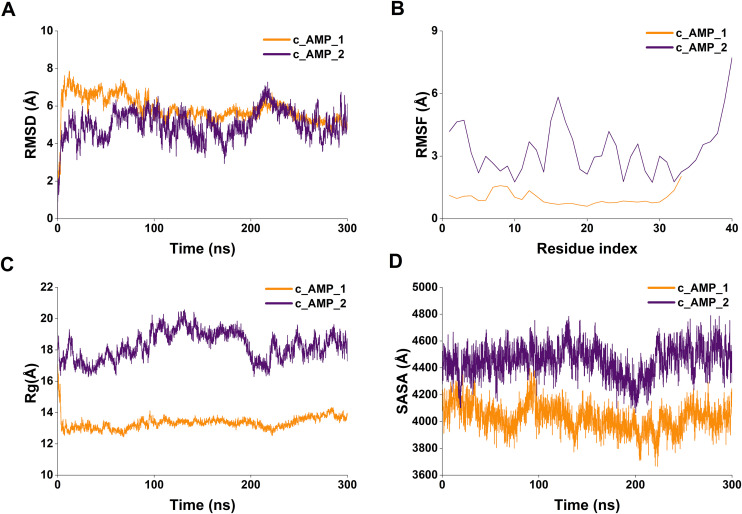
Structural dynamics of c_AMP_1 and c_AMP_2 in the *K. pneumoniae* membrane. (A) RMSD trajectories showing backbone stability over time. (B) RMSF plots reflecting residue-level flexibility. (C) SASA data illustrating how much of the peptide remained in contact with the surrounding solvent. (D) Rg profiles highlight the structural compactness of the peptides during simulations.

In the *K. pneumoniae* membrane, both peptides demonstrated comparable structural trends. c_AMP_1 stabilized with RMSD values around 1.3 Å and maintained these values throughout the simulation (Fig 3A). RMSF again revealed rigid core residues and flexible termini, peaking at 2.1 Å (Fig 3B). c_AMP_2 showed consistent dynamics, with RMSD ranging between 2.2 and 2.5 Å and higher C-terminal fluctuations (~3.1 Å), indicating continued flexibility in this membrane. SASA values followed the same pattern, with c_AMP_1 exposed (~48.7 nm²) and c_AMP_2 more buried (~30.1 nm²) (Fig 3C). Rg profiles remained stable for both peptides, averaging 1.35 nm for c_AMP_1 and 1.13 nm for c_AMP_2 (Fig 3D).

**Fig 4:**
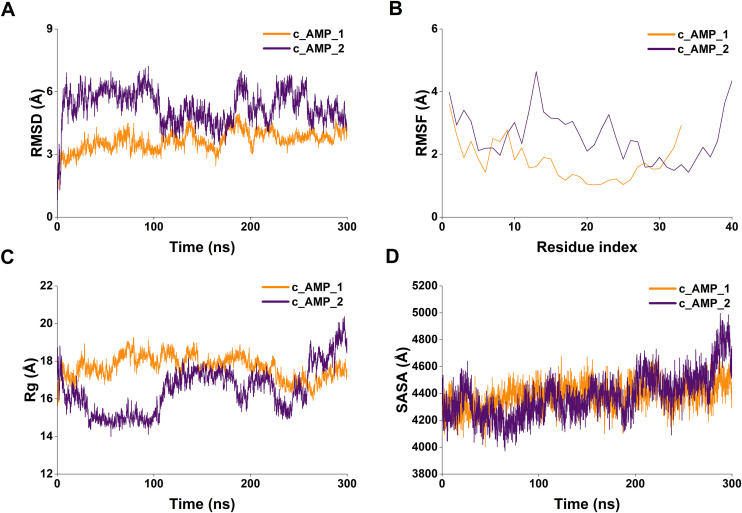
Structural dynamics of c_AMP_1 and c_AMP_2 in the *P. aeruginosa* membrane. (A) RMSD trajectories showing backbone stability over time. (B) RMSF plots reflecting residue-level flexibility. (C) SASA data illustrating how much of the peptide remained in contact with the surrounding solvent. (D) Rg profiles highlight the structural compactness of the peptides during simulations.

In the *P. aeruginosa* membrane, peptide dynamics were consistent with prior systems but slightly more restrained. c_AMP_1 reached RMSD equilibrium early and remained within 1.2–1.3 Å throughout (Fig 4A), with RMSF values under 1.0 Å in the helical core and a peak of 2.4 Å at the N-terminus (Fig 4B). c_AMP_2 displayed marginally reduced RMSD (2.1–2.4 Å) and RMSF fluctuations compared to other membranes, suggesting a slightly more stabilized configuration in this environment. SASA analysis again indicated c_AMP_1 was surface-oriented (~47.2 nm²), while c_AMP_2 was more shielded (~29.0 nm²) (Fig 4C). Rg values for c_AMP_1 ranged between 1.25 and 1.34 nm, while c_AMP_2 maintained its compact structure (~1.08–1.15 nm) (Fig 4D).

These analyses suggest that both peptides maintain structurally stable and compact conformations across all three bacterial membrane models, with c_AMP_2 exhibiting lower flexibility and solvent exposure compared to c_AMP_1. These predicted properties support their candidacy for further mechanistic evaluation of membrane interaction.

### 3.4. Peptide insertion kinetics and orientation in bacterial membranes

**Fig 5:**
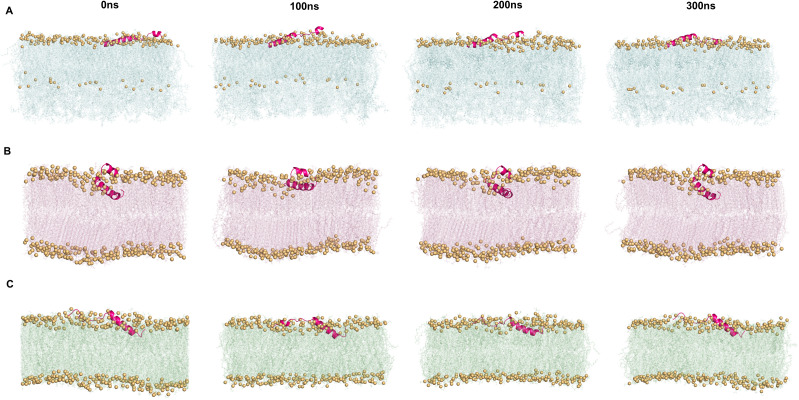
Representative MD snapshots showing the progressive membrane interaction of c_AMP_1 in bacterial systems. Snapshots captured at 0, 100, 200, and 300 ns illustrate the progressive association and partial embedding of c_AMP_1 within the lipid bilayers of (A) *A. baumannii*, (B) *K. pneumoniae*, and (C) *P. aeruginosa*.

**Fig 6:**
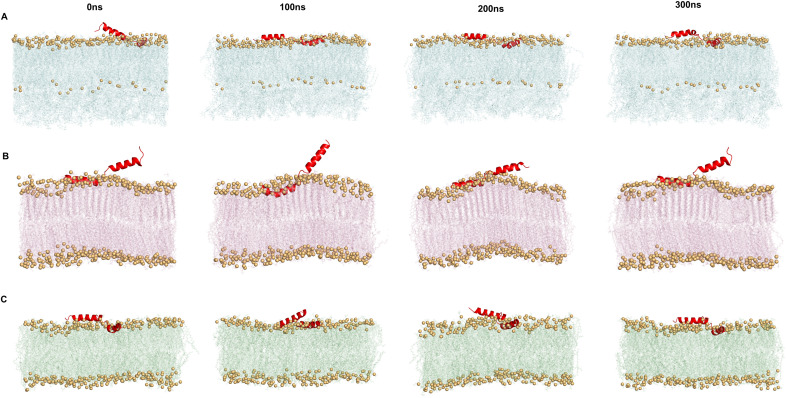
Representative MD snapshots illustrating rapid and stable membrane embedding of c_AMP_2 across bacterial systems. Snapshots at 0, 100, 200, and 300 ns highlight the swift insertion and persistent alignment of c_AMP_2 within the bilayers of (A) *A. baumannii*, (B) *K. pneumoniae*, and (C) *P. aeruginosa*.

To evaluate the dynamics of peptide-membrane interactions, molecular dynamics snapshots were extracted at 0, 100, 200, and 300 ns for c_AMP_1 and c_AMP_2 across three bacterial membranes, *A. baumannii*, *K. pneumoniae*, and *P. aeruginosa* (Fig 5 and Fig 6).

In all three bacterial membranes, c_AMP_1 demonstrated a gradual and surface-associated mode of interaction (Fig 5). At 0 ns, the peptide was positioned near the solvent interface. By 100 ns, partial alignment with the membrane surface was observed. At 200 ns, c_AMP_1 established persistent surface contact without deep insertion. By 300 ns, the peptide remained superficially embedded, aligning parallel to the membrane plane, particularly in *A. baumannii* and *K. pneumoniae*. In *P. aeruginosa*, limited penetration into the bilayer was observed, suggesting membrane composition may influence peptide orientation and depth.

c_AMP_2 exhibited markedly different behavior. In all three systems, it approached the membrane early and began embedding by 100 ns (Fig 6). At 200 ns, the helical segment was deeply inserted, and by 300 ns, the peptide adopted a transmembrane orientation in *K. pneumoniae* and a partially buried tilt in *P. aeruginosa*. In *A. baumannii*, it achieved the deepest insertion, where most of the hydrophobic core was fully embedded. This suggests that c_AMP_2 undergoes rapid membrane insertion and stabilizes within the bilayer interior.

**Fig 7:**
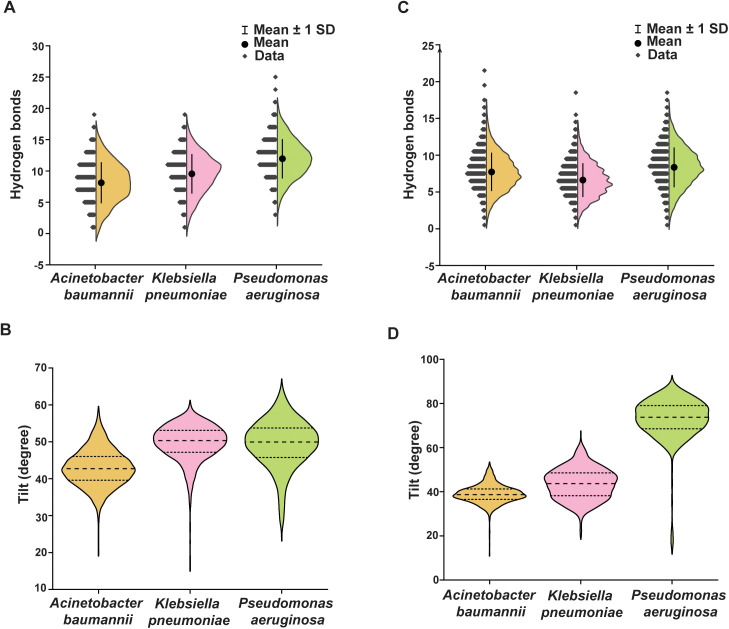
Analysis of hydrogen bonding patterns and angular orientation (tilt) of c_AMP_1 and c_AMP_2 across three different bacterial membrane environments. (A) Hydrogen bond counts of c_AMP_1 (B) Tilt angle distribution of c_AMP_1 (C) Hydrogen bond counts of c_AMP_2 (D) Tilt angle distribution of c_AMP_2 across *A. baumannii*, *K. pneumoniae*, and *P. aeruginosa*.

Quantitative analyses supported these observations. Hydrogen bond profiles revealed that c_AMP_1 formed a moderate number of lipid-peptide hydrogen bonds throughout the simulation, averaging ~2–4 per frame in *K. pneumoniae* and *P. aeruginosa* (Fig 7A). H-bonding was more frequent in *A. baumannii* (~6 per frame), suggesting stronger surface adherence in this membrane. These interactions were primarily mediated by lysine and arginine residues at the peptide termini, forming transient electrostatic contacts with the polar headgroups. Corresponding tilt angle distributions (Fig 7B) showed that c_AMP_1 consistently aligned between 75° and 90°, indicative of a parallel orientation along the membrane surface. The angle range was broader in *A. baumannii*, with occasional excursions toward 60°, reflecting minor orientation shifts but no stable insertion.

In contrast, c_AMP_2 established a significantly higher number of hydrogen bonds – averaging ~7–10 per frame across all membranes (Fig 7C). The strongest interaction was observed in *A. baumannii*, where c_AMP_2 maintained peak values between 8–12 H-bonds, while *K. pneumoniae* and *P. aeruginosa* exhibited slightly lower values (~6–8 per frame). These interactions were dominated by arginine and lysine residues engaging with lipid phosphate and glycerol groups, facilitating electrostatic stabilization. Tilt angle profiles (Fig 7D) revealed broader distributions for c_AMP_2, with values spanning 45° to 80°, reflecting a diagonally inserted orientation. The most stable and narrow angle range (~25°-35°) was observed in *A. baumannii*, supporting the observation of deeper insertion in this asymmetric membrane system. Together, the combination of high hydrogen bond frequency, sustained orientation, and membrane embedding suggests that c_AMP_2 may interact with the membrane more extensively than c_AMP_1 *in silico*, particularly in lipid environments with compositional asymmetry.

### 3.5. Residue-level interaction mapping and membrane perturbation analysis

**Fig 8:**
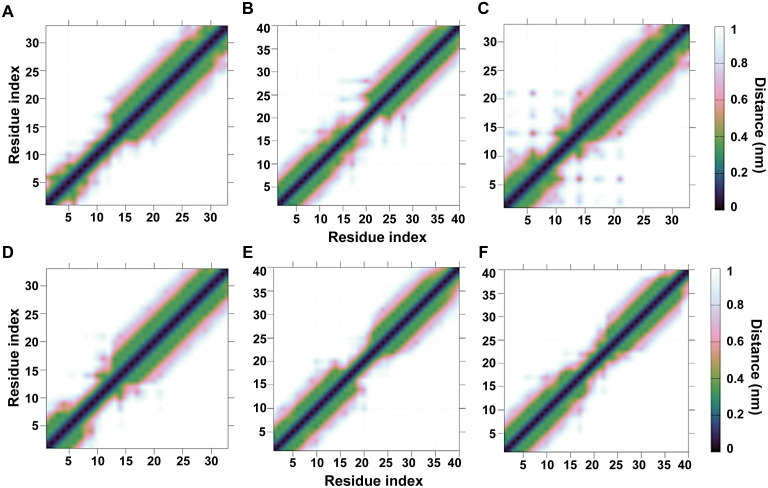
Residue-level contact maps of c_AMP_1 and c_AMP_2 across three bacterial membranes. Contact map of c_AMP_1 in (A) *A. baumannii*, (B) *K. pneumoniae*, and (C) *P. aeruginosa*; contact map of c_AMP_2 in (D) *A. baumannii*, (E) *K. pneumoniae*, and (F) *P. aeruginosa*.

To gain atomistic insights into how the peptides interact with bacterial membranes, residue-level contact maps were generated for c_AMP_1 and c_AMP_2 across all three systems (Fig 8). These maps visualize which amino acids maintain sustained proximity (<0.5 nm) to the membrane lipids over the 300 ns simulations.

For c_AMP_1, interaction hotspots were localized to a few basic residues at the N-terminal region, including Arg and Lys, especially in *A. baumannii* (Fig 8A) and *K. pneumoniae* (Fig 8B). However, in *P. aeruginosa* (Fig 8C), the contact density was notably reduced and fragmented, suggesting weaker and more transient peptide-lipid interactions. The limited and localized contact pattern aligns with previous observations of poor membrane embedding and low hydrogen bonding. In contrast, c_AMP_2 displayed widespread and persistent residue contacts spanning both the N- and C-terminal regions (Fig 8D-F). The key interacting residues included multiple lysines and arginines distributed along the peptide, supporting deep insertion and stable lipid engagement. In *A. baumannii* and *K. pneumoniae*, the contact intensity was high and continuous along the peptide backbone, suggesting strong and sustained electrostatic anchoring across the bilayer surface. Even in *P. aeruginosa*, where c_AMP_1 showed more limited and fragmented contacts, c_AMP_2 maintained broader interactions across the peptide, consistent with more extensive lipid engagement in this simulated system.

**Fig 9:**
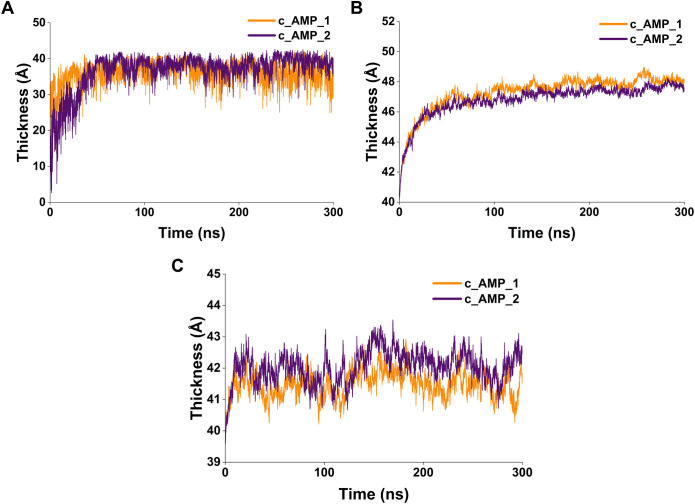
Membrane thickness profiles for c_AMP_1 and c_AMP_2 across bacterial membrane models. Thickness variations in (A) *A. baumannii*, (B) *K. pneumoniae*, and (C) *P. aeruginosa*.

**Fig 10:**
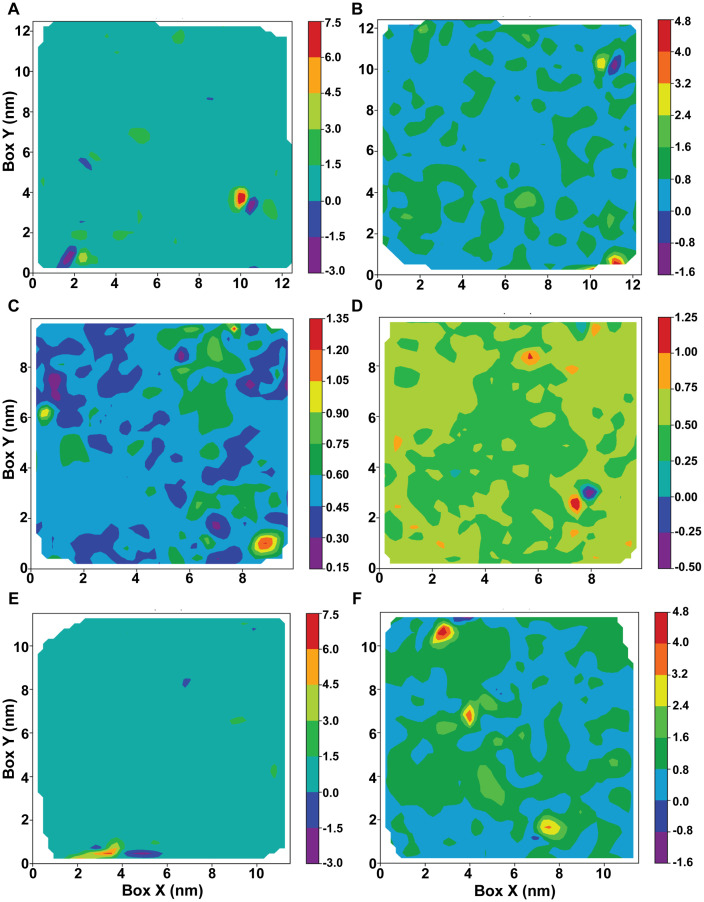
Area per lipid (APL) profiles of c_AMP_1 and c_AMP_2 across bacterial membranes. (A–C) APL distributions of c_AMP_1 in *A. baumannii*, *K. pneumoniae*, and *P. aeruginosa*, respectively. (D–F) APL distributions of c_AMP_2 in *A. baumannii*, *K. pneumoniae*, and *P. aeruginosa* respectively.

Membrane thickness maps further supported these interaction profiles (Fig 9). c_AMP_1 induced localized thinning beneath the peptide region in all three membranes (Fig 9A-C), with *A. baumannii* showing the most pronounced reduction (~1.1 Å), while *K. pneumoniae* and *P. aeruginosa* displayed only subtle deviations (<0.7 Å). These results are consistent with a surface-aligned conformation that perturbs the outer leaflet without significant penetration into the hydrophobic core. In contrast, c_AMP_2 induced notable bilayer deformation (Fig 9D-F), with thinning of ~2.1 Å in *A. baumannii*, ~1.8 Å in *K. pneumoniae*, and ~1.5 Å in *P. aeruginosa*. These reductions correspond to the high-contact zones seen in residue-level analysis, consistent with deeper insertion of c_AMP_2 and localized lipid compression within the simulated bilayers. Area per lipid (APL) analysis corroborated the extent of membrane perturbation caused by each peptide (Fig 10). c_AMP_1 resulted in modest APL expansion (Fig 10A-C), with a maximum increase of ~1.3% in *A. baumannii*, and less than 1% in *K. pneumoniae* and *P. aeruginosa*. This suggests minor adjustments in lateral lipid packing due to surface-level interaction. In contrast, c_AMP_2 led to significantly greater increases in APL across all membranes (Fig 10D-F), including ~3.2% in *A. baumannii*, ~2.4% in *K. pneumoniae*, and ~1.6% in *P. aeruginosa*. These data suggest that c_AMP_2 may perturb both vertical bilayer structure and lateral lipid organization to a greater extent than c_AMP_1 in the simulated systems, consistent with its deeper predicted insertion and stronger lipid engagement.

### 3.6. Secondary structure evolution during simulations

**Fig 11:**
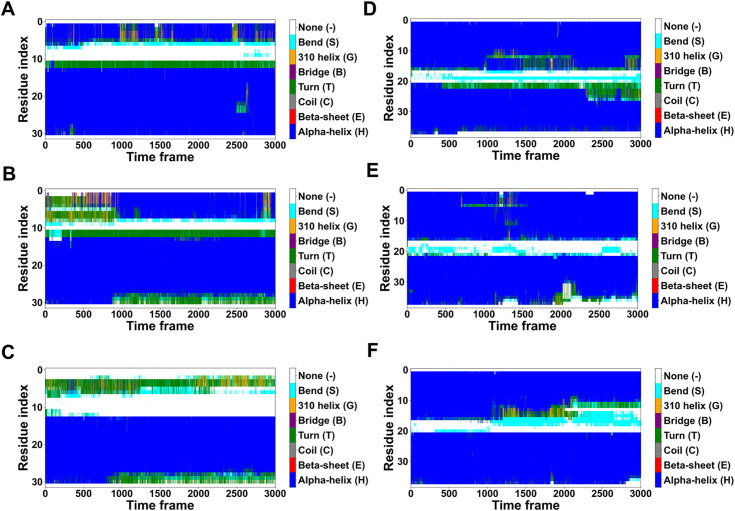
Secondary structure dynamics of c_AMP_1 and c_AMP_2 as revealed by DSSP throughout the course of the simulation. DSSP plots of c_AMP_1 in (A) *A. baumannii*, (B) *K. pneumoniae*, and (C) *P. aeruginosa*; DSSP plots of c_AMP_2 in (D) *A. baumannii*, (E) *K. pneumoniae*, and (F) *P. aeruginosa*.

The secondary structure dynamics of c_AMP_1 and c_AMP_2 were tracked throughout the 300 ns simulation to assess their structural integrity in membrane environments (Fig 11). Using DSSP analysis, we monitored the evolution of α-helicity and conformational transitions in *A. baumannii*, *K. pneumoniae*, and *P. aeruginosa* membranes.

c_AMP_1 maintained a stable α-helical conformation across all three systems, with minor fluctuations at the N- and C-terminal residues. In *A. baumannii*, the helical core (residues 10–30) was consistently preserved throughout the simulation, with transient unfolding events limited to terminal loops (Fig 11A). Similar trends were observed in *K. pneumoniae* and *P. aeruginosa* (Fig 11B and Fig 11C), where the core helix remained intact, and structural deviations were rare and short-lived. These results indicate that c_AMP_1 is structurally resilient in diverse membrane environments and adopts a conformation that supports surface-aligned interactions without significant destabilization.

In contrast, c_AMP_2 showed dynamic yet stable secondary structure behavior (Fig 11D-F). The central helix remained intact across all membranes, but conformational flexibility was evident in the terminal regions, particularly in *K. pneumoniae* (Fig 11E), where partial unwinding of the C-terminal end occurred after ~200 ns. Despite these fluctuations, the overall α-helical content remained high throughout the simulations. In *A. baumannii* (Fig 11D), the peptide retained a nearly continuous helical segment with minimal disruption, aligning with its deeper membrane insertion and stabilized orientation. *P. aeruginosa* (Fig 11F) exhibited slightly more flexible transitions in terminal regions but preserved the helical core, supporting sustained interaction with the bilayer. Collectively, these analyses suggest that both peptides preserve their secondary structure during simulated membrane engagement. c_AMP_1 appears rigid and helically stable, consistent with a surface-associated interaction mode, while c_AMP_2 displays predicted conformational flexibility that may facilitate deeper insertion and adaptive membrane interactions.

## 4. Discussion

The exploration of Indian marine microbiomes as a source of antimicrobial peptides has yielded significant and promising results that advance our understanding of natural peptide-based defenses in underexplored ecosystems. The identification of over 51,000 high-confidence c_AMPs through the integration of multiple machine learning tools reflects the value of ensemble prediction approaches in reducing false positives and enhancing prediction confidence. The stringency applied, which requires consensus across six independent classifiers, suggests that the shortlisted peptides are less likely to be artifacts of individual models and more likely to represent biologically plausible AMP candidates. This confidence is supported by the independent validation of each tool in its original publication; however, no additional in-study benchmarking against reference AMP datasets was performed, and this represents a limitation that future studies should address. This result is consistent with recent approaches to AMP discovery that emphasize high-throughput yet rigorously filtered pipelines, such as those employed by Santos-Júnior et al., who mined the global microbiome for AMPs using a deep learning approach and similarly reported that only approximately 0.2% of the predicted smORFs yielded confident AMP candidates [30]. The fact that a comparable high-confidence AMP yield (~0.21% of smORFs) was observed in Indian marine microbiomes underscores the untapped potential of this region’s biodiversity for bioactive compound discovery.

Further narrowing to ten membrane-penetrating c_AMPs based on physicochemical and structural criteria highlights the utility of incorporating known AMP features, such as cationicity, amphiphilicity, and residue composition (Arg and Trp), which are key determinants of membrane interaction [57]. These features are widely recognized in natural AMPs, such as LL-37 and magainin, for their ability to disrupt bacterial membranes [58]. Notably, the strong net charges and isoelectric points (>10) observed in peptides such as c_AMP_1 (net charge +11, pI 12.02) suggest robust electrostatic attraction to negatively charged bacterial membranes, particularly those of Gram-negative species such as *A. baumannii* and *P. aeruginosa*. The elevated amphiphilicity indices of several peptides (e.g., c_AMP_1: 1.59; c_AMP_5: 1.75) further suggest favourable insertion into lipid bilayers and subsequent disruption of membrane integrity.

It should be noted, however, that the application of a length cut-off of ≤50 amino acids and the requirement for both arginine and tryptophan residues inherently narrows the candidate set toward cationic, membrane-active peptides. This filter, while effective for identifying membrane-targeting candidates, may inadvertently exclude other biologically relevant AMP classes such as anionic peptides, proline-rich AMPs, or peptides that act through intracellular mechanisms. This represents a limitation of the current pipeline, and future studies may benefit from broader compositional filters to capture the full diversity of AMP types present in marine metagenomic datasets.

The *in silico* safety profiling of the ten shortlisted peptides using ToxinPred 3.0 and HemoPI 2.0 revealed that all candidates were predicted as non-toxic, while hemolytic activity predictions indicated variable but moderate predicted HC50 values ranging from 18.3 to 126.4 μM. While these results suggest a potentially acceptable safety window, particularly for c_AMP_2 and c_AMP_7 which showed lower predicted hemolytic potency, *in silico* hemolysis predictions have inherent limitations and experimental validation through red blood cell hemolysis assays and mammalian cytotoxicity testing remains essential before any safety conclusions can be drawn.

AlphaFold-based structural predictions provided crucial insights into the 3D conformations of the selected peptides, particularly c_AMP_1 and c_AMP_2. The predominance of α-helical structures in c_AMP_1 is in line with canonical membrane-active peptides, which often adopt such conformations upon interaction with lipid bilayers [59]. The helical wheel diagrams displayed a typical amphipathic pattern, with hydrophobic amino acids grouped on one side of the helix and positively charged residues on the opposite side, an arrangement that is consistent with membrane association and interaction in bacterial cells. Despite being only partially helical, c_AMP_2 maintained its amphipathic nature, which is crucial for membrane engagement. This suggests that a combination of incomplete helicity and distinct charge distribution can still promote effective membrane interaction, an observation consistent with earlier reports on functionally active yet structurally flexible AMPs [60].

Molecular dynamics simulations in three membrane environments representing *P. aeruginosa*, *A. baumannii*, and *K. pneumoniae* revealed important dynamics of peptide-membrane interactions. c_AMP_1 displayed rapid stabilization (RMSD ~1.2–1.3 Å), particularly in *P. aeruginosa*, which is indicative of conformational rigidity and persistent structural integrity during membrane engagement. Its low RMSF values across the core helix further support structural stability, while increased SASA implies surface localization with effective membrane binding rather than deep insertion. In contrast, c_AMP_2 exhibited higher RMSD (2.2–2.5 Å) and RMSF, especially in the C-terminal region, reflecting greater flexibility and mobility within the bilayer. Its lower SASA values suggest deeper embedding into the membrane, likely due to its more compact conformation and hydrophobic distribution. These contrasting behaviors illustrate the distinct membrane interaction modes the two peptides may adopt, ranging from surface-associated orientation to deeper membrane embedding, both of which have been documented in membrane-active peptides such as melittin and indolicidin [61–63].

The hydrogen bonding profiles between the peptides and the lipid membranes further corroborate these distinct interaction patterns. c_AMP_1 formed ~2–6 hydrogen bonds per frame, with peak values in *A. baumannii* (~6/frame), supporting its stable yet superficial interaction with the membrane headgroups. These interactions predominantly involve arginine and lysine side chains engaging with phosphate or carbonyl oxygens of lipid headgroups, a mechanism also observed in other high-charge AMPs, such as defensins [64–66]. Although c_AMP_2 formed a consistently higher number of H-bonds (~7–12/frame), dominated by Arg and Lys sidechains, it maintained stable contacts deeper in the bilayer, suggesting that van der Waals and hydrophobic interactions play a greater role in its anchoring and membrane embedding.

Peptide tilt angles relative to the membrane normal provided insights into peptide orientation states during embedding. c_AMP_1 showed a consistent tilt range of ~75°-90°, indicating parallel surface alignment, which is consistent with a surface-associated orientation reminiscent of carpet-like interaction models. c_AMP_2 showed broader tilt values (~45°-80°), suggesting a more deeply embedded, diagonal orientation reminiscent of insertion-type interaction models observed in longer or more hydrophobic AMPs [13]. The observed angles also varied slightly between the membranes, with the greatest tilting occurring in the *K. pneumoniae* system, indicating that lipid composition influences peptide orientation and possibly efficacy.

An important consideration in interpreting the cross-species simulation results is that the three membrane models employed in this study are not architecturally equivalent. The *A. baumannii* model incorporates an asymmetric outer membrane containing Lipid A/LPS, whereas the *K. pneumoniae* and *P. aeruginosa* models represent symmetric inner membrane phospholipid bilayers lacking LPS. LPS is known to exert a significant influence on peptide binding, insertion depth, and membrane perturbation through its bulky polysaccharide chains and strong electrostatic interactions with cationic peptides (Jiang et al., 2020; Gong et al., 2023). Consequently, the observed differences in peptide behavior across the three membrane systems reflect both species-specific lipid compositions and the fundamentally different membrane architectures modelled. Direct quantitative comparisons of interaction metrics across systems should therefore be interpreted with caution, and this represents a limitation of the current comparative analysis. Future studies employing equivalent outer membrane models incorporating species-specific LPS compositions for all three organisms would enable more rigorous cross-species comparisons.

Residue-lipid contact maps further revealed that for c_AMP_1, arginine and tryptophan residues were key in anchoring the peptide to membrane headgroups, forming consistent contacts over the simulation period. This preference for cation-π and hydrogen bonding interactions with phospholipid headgroups reflects conserved AMP behavior, where these residues facilitate initial binding and destabilization [67–69]. In contrast, c_AMP_2 exhibited a broader interaction footprint, engaging mid-helix residues with the lipid tail region, pointing to a more deeply embedded orientation in the simulations; whether this translates into actual membrane permeabilization cannot be determined from these structural simulations alone and would require dedicated experimental assays.

Membrane thickness and area per lipid analyses revealed biophysical changes induced by peptide interactions. c_AMP_1 caused mild thinning (~1.1 Å) beneath the contact zone in *A. baumannii*, and <0.7 Å in *K. pneumoniae* and *P. aeruginosa*. This result aligns with its higher SASA and lower tilt. c_AMP_2 induced more pronounced thinning (~2.1 Å in *A. baumannii*, ~ 1.8 Å in *K. pneumoniae*) and increased the area per lipid by ~3.2% in *A. baumannii*, ~ 2.4% in *K. pneumoniae*, and ~1.6% in *P. aeruginosa*. These metrics mirror findings from MD simulations of other membrane-active AMPs, such as MSI-594, which induce membrane thinning and lateral expansion as part of their predicted interaction profile [13,70].

Secondary structure evolution using DSSP during the simulations revealed that c_AMP_1 showed partial helical loss, especially in *P. aeruginosa*, where helicity declined after ~150 ns, underscoring its conformational rigidity and structural resilience, which are critical for sustained membrane interaction. c_AMP_2 retained >80% helical content across all membranes, with minor terminal flexibility in *K. pneumoniae* and *P. aeruginosa* with ~65–70% helical content interspersed with coil and turn motifs, especially at terminal regions. This plasticity may facilitate membrane penetration and adaptation to various lipid environments, as observed in structurally flexible AMPs such as protegrins [71]. Collectively, these structural dynamics highlight the predicted biophysical divergence between the two peptides, suggesting they may adopt distinct membrane interaction modes, pending experimental validation.

While this study presents a comprehensive *in silico* characterization of candidate AMPs, the absence of experimental validation remains a notable limitation. Future studies should prioritize wet-lab validation of the lead candidates, c_AMP_1 and c_AMP_2, through: (i) minimum inhibitory concentration (MIC) assays against representative ESKAPE strains, (ii) membrane permeabilization assays using fluorescent indicators such as SYTOX Green or propidium iodide, (iii) liposome-based membrane leakage assays to confirm bilayer disruption, and (iv) hemolysis and cytotoxicity assays on mammalian cell lines such as RBCs and HEK293 to evaluate selectivity and safety. These experiments will be essential to confirm the predicted antimicrobial potential identified through computational analyses.

Several inherent limitations of the single-peptide MD simulations employed in this study warrant explicit acknowledgement. While the 300 ns all-atom simulations provide detailed insights into geometric preferences, orientation states, and lipid perturbation patterns of individual peptides, they cannot, in isolation, establish functional outcomes such as pore formation, membrane permeabilization, or bactericidal activity. Single-peptide simulations do not capture the cooperative behavior of multiple peptides interacting simultaneously with a membrane, which is often a prerequisite for functional membrane disruption. Furthermore, each simulation system was run as a single trajectory, which limits statistical confidence, and the 300 ns timescale, while extensive for single-peptide systems, may not fully capture slow membrane reorganization events, particularly in the asymmetric LPS-containing outer membrane model of *A. baumannii*, where lipid rearrangement and peptide-induced perturbations may occur on longer timescales than those sampled here. Future studies employing multiple replicates or enhanced sampling methods would provide stronger statistical support and deeper insights into these dynamics. Consequently, all observations from the MD simulations in this study are interpreted as predicted interaction modes and biophysical characterization rather than as demonstrated antimicrobial mechanisms, and experimental validation remains essential before any functional claims can be made.

It is important to acknowledge that the machine learning tools employed in this study were primarily trained on general AMP datasets, which may underrepresent experimentally validated antitubercular peptides, potentially limiting the sensitivity of the pipeline for Mycobacterium-active peptides. While the primary focus of this study is on Gram-negative ESKAPE pathogens, an exploratory screening of the ten shortlisted candidates using AntiTbPred [72] revealed that all ten peptides were predicted as potential anti-tubercular peptides, with prediction scores ranging from 0.26 to 1.72 (**Table E in** **S1 File**). These findings are presented as exploratory observations and should not be interpreted as confirmed anti-mycobacterial activity, which would require dedicated experimental validation including MIC assays against Mycobacterium tuberculosis strains. Future studies are recommended to benchmark the pipeline against specialized resources such as AntiTbPdb [73] to more rigorously assess its sensitivity for anti-mycobacterial peptide discovery.

To contextualize the translational potential of the lead candidates, similarity searches were performed against THPdb2 [74], a curated database of FDA-approved therapeutic peptides and proteins. No statistically significant sequence similarity was detected for either c_AMP_1 (E-value: 1.7) or c_AMP_2 (E-value: 6.2), confirming their structural distinctiveness from currently approved therapeutics (**Table F in** **S1 File**). Notably, both candidates fall within the ‘Long Peptides (21–50 AA)’ category of THPdb2, which encompasses 45 unique FDA-approved therapeutic peptides, indicating that their length profile is consistent with clinically approved peptide therapeutics. Furthermore, their strongly cationic nature, amphipathic architecture, and predicted membrane-targeting properties align well with the physicochemical characteristics associated with approved antimicrobial peptide therapeutics. Collectively, these observations indicate that c_AMP_1 and c_AMP_2 possess physicochemical attributes broadly consistent with those of known membrane-active and clinically approved peptides. In the absence of experimental data, however, these similarities should be regarded as preliminary, and the two peptides are best viewed as computational starting points that will require extensive experimental validation before any therapeutic potential can be established.

## 5. Conclusion

Over 51,000 high-confidence c_AMPs were identified from Indian marine microbiomes using a consensus-based approach across six machine learning classifiers, suggesting a reduction in the number of false positives. Ten membrane-active c_AMPs were shortlisted on the basis of key properties, such as their cationicity, amphiphilicity, and net positive charge, which are essential for bacterial membrane targeting. Structural predictions revealed amphipathic α-helices in top peptides such as c_AMP_1, supporting their predicted potential for membrane insertion and perturbation *in silico*. MD simulations revealed distinct membrane interaction modes: c_AMP_1 remained surface-associated, while c_AMP_2 adopted a more deeply embedded orientation, suggesting distinct biophysical interaction profiles. The peptides induced membrane thinning and area expansion, especially c_AMP_2, suggesting predicted biophysical perturbation of the simulated bacterial membrane models. c_AMP_1 exhibited stable α-helicity and a low RMSD, whereas c_AMP_2 showed flexible secondary structures, indicating adaptability to different membrane environments. Arginine and tryptophan residues are predicted to play important roles in membrane binding and anchoring in the simulated systems, which is consistent with established AMP interaction models. Taken together, these computational findings point to the predicted membrane-active potential of marine-derived candidate AMPs against Gram-negative ESKAPE pathogens and underscore the need for experimental validation of their antimicrobial activity and safety, including MIC assays, membrane permeabilization studies, and cytotoxicity testing in relevant models, before any functional or therapeutic conclusions can be drawn.

## Supporting information

S1 File Supporting tables and figures referenced in the main text. (DOCX)

S2 File List of high-confidence antimicrobial peptides (c_AMPs) predicted from Indian marine metagenomic datasets. (ZIP)
