# Supplementary material for: In silico identification and biophysical characterization of candidate antimicrobial peptides from the Indian marine microbiome targeting multidrug-resistant ESKAPE pathogens
Source: PLoS One. 2026 Jul 22;21(7):e0353985. doi: 10.1371/journal.pone.0353985 (PMC13390849; doi:10.1371/journal.pone.0353985)
Supplement: S1 File — (DOCX) [file pone.0353985.s001.docx]

**Supplementary Information**

***In silico* identification and biophysical characterization of candidate antimicrobial peptides from the Indian marine microbiome targeting multidrug-resistant ESKAPE pathogens**

Sreelakshmi K V^1^, Nasri Thaha^1^ and Budheswar Dehury^1*^

^1^Department of Bioinformatics, Manipal School of Life Sciences, Manipal Academy of

Higher Education, Manipal – 576104, Karnataka, India

^*^**Corresponding Author**

Dr. Budheswar Dehury

Email: budheswar.dehury@manipal.edu, [budheswar.dehury@gmail.com](mailto:budheswar.dehury@gmail.com)

**Table of Contents**

| **Sl No.** | **Caption** | **Page No.** |
| --- | --- | --- |
| **Table A** | Metadata of selected Indian marine metagenomic BioProjects used in this study. | S3 |
| **Table B** | Read counts and sequence retention across processing steps for Indian marine metagenomic datasets. | S7 |
| **Table C** | Cross-validation of shortlisted candidate antimicrobial peptides using machine learning-based CPP prediction tools. | S8 |
| **Table D** | CPPsite 3.0 similarity search results of the ten shortlisted candidate antimicrobial peptides against the curated CPP database. | S9 |
| **Table E** | AntiTbPred prediction results of the ten shortlisted candidate antimicrobial peptides for exploratory anti-mycobacterial activity assessment. | S10 |
| **Table F** | THPdb2 similarity search results for lead candidate antimicrobial peptides c_AMP_1 and c_AMP_2 against the curated database of FDA-approved therapeutic peptides and proteins. | S11 |
| **Table G** | *In silico* toxicity and hemolytic activity predictions of the ten shortlisted candidate antimicrobial peptides using ToxinPred 3.0 and HemoPI 2.0. | S12 |
| **Fig. A** | Predicted three-dimensional structures of the top 10 cell-penetrating c_AMPs. | S13 |
| **Fig. B** | Helical wheel diagrams of the 10 selected cell-penetrating c_AMPs. | S14 |

**Table A. Metadata of selected Indian marine metagenomic BioProjects used in this study.**
This table provides detailed metadata for the BioSamples included in the study, including BioProject accession numbers, BioSample IDs, SRA study links, geographic locations, sequencing platforms, source organisms or environments, and sampling coordinates (latitude and longitude).

| **BioProject** | **BioSample** | **SRA Study** | **Location** | **Platform** | **Organism** | **lat_lon** |
| --- | --- | --- | --- | --- | --- | --- |
| **PRJNA822508** | SAMN27189576 | SRP367105 | Andaman and Nicobar Islands | ILLUMINA | seawater metagenome | 11.9845 N 92.9508 E |
| **PRJNA822508** | SAMN27189575 | SRP367105 | Andaman and Nicobar Islands | ILLUMINA | seawater metagenome | 12.0081 N 92.9416 E |
| **PRJNA822508** | SAMN27189573 | SRP367105 | Andaman and Nicobar Islands | ILLUMINA | seawater metagenome | 11.4031 N 92.4547 E |
| **PRJNA822508** | SAMN27189578 | SRP367105 | Andaman and Nicobar Islands | ILLUMINA | seawater metagenome | 11.5060 N 92.7015 E |
| **PRJNA822508** | SAMN27189577 | SRP367105 | Andaman and Nicobar Islands | ILLUMINA | seawater metagenome | 11.4019 N 92.4455 E |
| **PRJNA822508** | SAMN27189574 | SRP367105 | Andaman and Nicobar Islands | ILLUMINA | seawater metagenome | 11.96 N 93.00 E |
| **PRJNA891635** | SAMN31340263 | SRP403200 | Arabian sea | ILLUMINA | seawater metagenome | 15.24 N 73.36 E |
| **PRJNA891635** | SAMN31340261 | SRP403200 | Laccadive Sea | ILLUMINA | seawater metagenome | 8.28 N 76.51 E |
| **PRJNA891635** | SAMN31340260 | SRP403200 | Laccadive Sea | ILLUMINA | seawater metagenome | 12.50 N 73.56 E |
| **PRJNA891635** | SAMN31340259 | SRP403200 | Laccadive Sea | ILLUMINA | seawater metagenome | 12.50 N 74.40 E |
| **PRJNA891635** | SAMN31340268 | SRP403200 | Laccadive Sea | ILLUMINA | seawater metagenome | 8.30 N 76.13 E |
| **PRJNA891635** | SAMN31340267 | SRP403200 | Laccadive Sea | ILLUMINA | seawater metagenome | 8.28 N 76.51 E |
| **PRJNA891635** | SAMN31340258 | SRP403200 | Arabian sea | ILLUMINA | seawater metagenome | 15.23 N 72.46 E |
| **PRJNA891635** | SAMN31340257 | SRP403200 | Arabian sea | ILLUMINA | seawater metagenome | 15.24 N 73.36 E |
| **PRJNA891635** | SAMN31340265 | SRP403200 | Laccadive Sea | ILLUMINA | seawater metagenome | 12.50 N 74.40 E |
| **PRJNA891635** | SAMN31340266 | SRP403200 | Laccadive Sea | ILLUMINA | seawater metagenome | 12.50 N 73.56 E |
| **PRJNA891635** | SAMN31340264 | SRP403200 | Arabian sea | ILLUMINA | seawater metagenome | 15.23 N 72.46 E |
| **PRJNA891635** | SAMN31340262 | SRP403200 | Laccadive Sea | ILLUMINA | seawater metagenome | 8.30 N 76.13 E |
| **PRJNA900060** | SAMN36399917 | SRP409134 | Valinokkam | ILLUMINA | sponge metagenome | 9.1841 N 78.6370 E |
| **PRJNA900060** | SAMN36399916 | SRP409134 | Maraikyarpattinam | ILLUMINA | sponge metagenome | 9.2724 N 79.1287 E |
| **PRJNA900060** | SAMN36399915 | SRP409134 | Maraikayar Pattinam | ILLUMINA | sponge metagenome | 9.2724 N 79.1287 E |
| **PRJNA900060** | SAMN36399914 | SRP409134 | Pamban Bridge | ILLUMINA | sediment metagenome | 9.2825 N 79.1933 E |
| **PRJNA900060** | SAMN36399913 | SRP409134 | Pudumadam | ILLUMINA | algae metagenome | 9.2771 N 78.9939 E |
| **PRJNA900060** | SAMN36399912 | SRP409134 | Pudumadam | ILLUMINA | algae metagenome | 9.2771 N 78.9939 E |
| **PRJNA900060** | SAMN36399911 | SRP409134 | Pudumadam | ILLUMINA | algae metagenome | 9.2771 N 78.9939 E |
| **PRJNA900060** | SAMN36399910 | SRP409134 | Vattakottai Fort | ILLUMINA | algae metagenome | 9.1841 N 78.6370 E |
| **PRJNA900060** | SAMN36399919 | SRP409134 | Pudumadam | ILLUMINA | sponge metagenome | 9.2771 N 78.9939 E |
| **PRJNA900060** | SAMN36399918 | SRP409134 | Pudumadam | ILLUMINA | sponge metagenome | 9.2771 N 78.9939 E |
| **PRJNA900060** | SAMN36399909 | SRP409134 | Pudumadam | ILLUMINA | algae metagenome | 9.2771 N 78.9939 E |
| **PRJNA900060** | SAMN36399908 | SRP409134 | Pudumadam | ILLUMINA | algae metagenome | 9.2771 N 78.9939 E |
| **PRJNA900060** | SAMN43521959 | SRP409134 | Pudumadam | ILLUMINA | algae metagenome | 9.2731 N 78.9908 E |
| **PRJNA900060** | SAMN31850539 | SRP409134 | Rameshwaram | ILLUMINA | algae metagenome | 9.2876 N 79.3129 E |
| **PRJNA900060** | SAMN31853059 | SRP409134 | Maraikayarpattinam | ILLUMINA | marine metagenome | 9.2724 N 79.1287 E |
| **PRJNA900060** | SAMN32146946 | SRP409134 | Rameshwaram | ILLUMINA | algae metagenome | 9.2876 N 79.3129 E |
| **PRJNA900060** | SAMN31846935 | SRP409134 | Rameshwaram | ILLUMINA | algae metagenome | 9.2876 N 79.3129 E |
| **PRJNA900060** | SAMN31848137 | SRP409134 | Rameshwaram | ILLUMINA | algae metagenome | 9.2876 N 79.3129 E |
| **PRJNA900060** | SAMN31831536 | SRP409134 | Manapad beach | ILLUMINA | algae metagenome | 8.3752 N 78.0637 E |
| **PRJNA900060** | SAMN31891915 | SRP409134 | Manapad beach | ILLUMINA | algae metagenome | 8.3752 N 78.0637 E |
| **PRJNA900060** | SAMN31846934 | SRP409134 | Maraikayar Pattinam | ILLUMINA | sediment metagenome | 9.2724 N 79.1287 E |
| **PRJNA900060** | SAMN44959751 | SRP409134 | Vattakottai Fort | DNBSEQ | algae metagenome | 8.125 N 77.565 E |
| **PRJNA900060** | SAMN45199050 | SRP409134 | Manapad | DNBSEQ | algae metagenome | 8.37302 N 78.06496 E |
| **PRJNA928230** | SAMN32925254 | SRP419214 | Palkbay | ILLUMINA | coral metagenome | 9.287944 N 79.215250 E |
| **PRJNA928230** | SAMN32925253 | SRP419214 | Palkbay | ILLUMINA | coral metagenome | 9.287944 N 79.215250 E |
| **PRJNA928230** | SAMN32925252 | SRP419214 | Palkbay | ILLUMINA | coral metagenome | 9.292417 N 79.218306 E |
| **PRJNA928230** | SAMN32925251 | SRP419214 | Palkbay | ILLUMINA | coral metagenome | 9.292417 N 79.218306 E |
| **PRJNA928230** | SAMN32925250 | SRP419214 | Palkbay | ILLUMINA | coral metagenome | 9.292417 N 79.218306 E |
| **PRJNA928230** | SAMN32925249 | SRP419214 | Palkbay | ILLUMINA | coral metagenome | 9.292417 N 79.218306 E |
| **PRJNA928230** | SAMN32925248 | SRP419214 | Palkbay | ILLUMINA | coral metagenome | 9.290222 N 79.216389 E |
| **PRJNA928230** | SAMN32925247 | SRP419214 | Palkbay | ILLUMINA | coral metagenome | 9.292417 N 79.218306 E |
| **PRJNA928230** | SAMN32925257 | SRP419214 | Palkbay | ILLUMINA | coral metagenome | 9.290222 N 79.216389 E |
| **PRJNA928230** | SAMN32925256 | SRP419214 | Palkbay | ILLUMINA | coral metagenome | 9.287944 N 79.215250 E |
| **PRJNA928230** | SAMN32925255 | SRP419214 | Palkbay | ILLUMINA | coral metagenome | 9.287944 N 79.215250 E |
| **PRJNA928230** | SAMN32925246 | SRP419214 | Palkbay | ILLUMINA | coral metagenome | 9.290222 N 79.216389 E |
| **PRJNA928230** | SAMN32925245 | SRP419214 | Palkbay | ILLUMINA | marine metagenome | 9.290222 N 79.216389 E |
| **PRJNA971765** | SAMN43517068 | SRP444064 | Gulf of Mannar | ILLUMINA | sediment metagenome | 8.70 N 78.10 E |
| **PRJNA971765** | SAMN43517069 | SRP444064 | Gulf of Mannar | ILLUMINA | sponge metagenome | 9.27 N 79.00 E |
| **PRJNA971765** | SAMN43517070 | SRP444064 | Gulf of Mannar | ILLUMINA | algae metagenome | 9.27 N 79.00 E |
| **PRJNA971765** | SAMN43517071 | SRP444064 | Gulf of Mannar | ILLUMINA | algae metagenome | 9.27 N 79.00 E |
| **PRJNA971765** | SAMN43517072 | SRP444064 | Gulf of Mannar | ILLUMINA | algae metagenome | 9.27 N 79.00 E |

**Table B. Read counts and sequence retention across processing steps for Indian marine metagenomic datasets.** Summary of raw and trimmed read counts, assembled contigs, predicted ORFs and smORFs, non-redundant smORFs, and the number of high-confidence AMPs commonly predicted by all six machine learning tools across five marine BioProjects. (M-million reads)

| BioProject | Raw Read Count (M) | Read Count (After trimming) (M) | Contigs(M) | ORFs (M) | smORFs (33-450 bp) (M) | Non-redundant smORFs (M) | Commonly predicted AMPs |
| --- | --- | --- | --- | --- | --- | --- | --- |
| PRJNA822508 | 58.3 | 44.4 | 1.7 | 1.68 | 1.53 | 1.39 | 2,915 |
| PRJNA891635 | 80.8 | 55.3 | 6.7 | 7.6 | 6.5 | 4.9 | 8,937 |
| PRJNA900060 | 523.6 | 467.4 | 13.6 | 16.6 | 13.1 | 11.4 | 21,394 |
| PRJNA928230 | 166.6 | 158.2 | 8.1 | 6.1 | 5.5 | 4.3 | 15,773 |
| PRJNA971765 | 96.3 | 69 | 2.5 | 3.3 | 2.4 | 2.2 | 2,175 |

**Table C: Cross-validation of shortlisted candidate antimicrobial peptides using machine learning-based CPP prediction tools.** This table summarizes the CPP prediction outcomes for each candidate peptide, including the peptide ID, sequence, length, and classification results from pLM4CPPs and CellPPD-Mod prediction tools, along with the consensus prediction.

| **ID** | **Sequence** | **Length** | **pLM4CPPs Prediction** | **CellPPD Prediction** |
| --- | --- | --- | --- | --- |
| c_AMP_1 | RWVAKRTRKFPRKYTQVAKKKTLLARLILYLIG | 33 | CPP | CPP |
| c_AMP_2 | GHDEAKAFMTCGLAGKRGGKAPRRWQHLGNMLNRLLSCRS | 40 | CPP | CPP |
| c_AMP_3 | FWLLGRWLRGLWRKRKAEQAAS | 22 | CPP | CPP |
| c_AMP_4 | DLGIRTAEKLEKKIRWFIKGRKAVKKLFEKEARHLNCF | 38 | CPP | CPP |
| c_AMP_5 | RWVSKRIRKFPRKYKHILRKTILYFIGS | 28 | CPP | CPP |
| c_AMP_6 | GSSFLKGGLCGRKSGGLLQVLQRWIKG | 27 | CPP | Non-CPP |
| c_AMP_7 | KKAARDHKRWWQVARHTARLIVGSAA | 26 | CPP | CPP |
| c_AMP_8 | REYGKRLRTGNAPLLLTGGVLALAGLLGGKRGWRRWARLALIVAPLLRRR | 49 | CPP | CPP |
| c_AMP_9 | LSRPPDVGMRWKWVLAAAAAKAALCGWHTTLLQAKTKAATLV | 42 | CPP | CPP |
| c_AMP_10 | PRWMRRFNRGMALLLLLSAWAAAFW | 25 | CPP | CPP |

**Table D: CPPsite 3.0 similarity search results of the ten shortlisted candidate antimicrobial peptides against the curated CPP database.** This table presents the Smith-Waterman alignment results for each candidate peptide, including the peptide ID, sequence, length, best alignment length in residues, percentage identity, and alignment score against known cell-penetrating peptides in the CPPsite 3.0 database.

| **ID** | **Sequence** | | **Length** | **CPPsite 3.0 Alignment Length (residues)** | **CPPsite 3.0 Identity (%)** | **CPPsite 3.0 Score** |
| --- | --- | --- | --- | --- | --- | --- |
| c_AMP_1 | RWVAKRTRKFPRKYTQVAKKKTLLARLILYLIG | | 33 | 7 | 42.9 | 17.0 |
| c_AMP_2 | GHDEAKAFMTCGLAGKRGGKAPRRWQHLGNMLNRLLSCRS | 40 | | 6 | 50.0 | 16.0 |
| c_AMP_3 | FWLLGRWLRGLWRKRKAEQAAS | | 22 | 7 | 28.6 | 16.0 |
| c_AMP_4 | DLGIRTAEKLEKKIRWFIKGRKAVKKLFEKEARHLNCF | | 38 | 3 | 100.0 | 16.0 |
| c_AMP_5 | RWVSKRIRKFPRKYKHILRKTILYFIGS | | 28 | 7 | 42.9 | 15.0 |
| c_AMP_6 | GSSFLKGGLCGRKSGGLLQVLQRWIKG | | 27 | 3 | 100.0 | 16.0 |
| c_AMP_7 | KKAARDHKRWWQVARHTARLIVGSAA | | 26 | 9 | 33.3 | 17.0 |
| c_AMP_8 | REYGKRLRTGNAPLLLTGGVLALAGLLGGKRGWRRWARLALIVAPLLRRR | | 49 | 6 | 66.7 | 16.0 |
| c_AMP_9 | LSRPPDVGMRWKWVLAAAAAKAALCGWHTTLLQAKTKAATLV | | 42 | 3 | 100.0 | 19.0 |
| c_AMP_10 | PRWMRRFNRGMALLLLLSAWAAAFW | | 25 | 8 | 50.0 | 13.0 |

**Table E: AntiTbPred prediction results of the ten shortlisted candidate antimicrobial peptides for exploratory anti-mycobacterial activity assessment.** This table presents the AntiTbPred prediction scores and classification outcomes for each candidate peptide, including the peptide ID, sequence, length, prediction score, and predicted activity.

| **ID** | **Sequence** | **Length** | **AntiTbPred Score** | **Prediction** |  |
| --- | --- | --- | --- | --- | --- |
| c_AMP_1 | RWVAKRTRKFPRKYTQVAKKKTLLARLILYLIG | 33 | 0.90 | Anti-tubercular peptide |  |
| c_AMP_2 | GHDEAKAFMTCGLAGKRGGKAPRRWQHLGNMLNRLLSCRS | 40 | 0.59 | Anti-tubercular peptide |  |
| c_AMP_3 | FWLLGRWLRGLWRKRKAEQAAS | 22 | 1.62 | Anti-tubercular peptide |  |
| c_AMP_4 | DLGIRTAEKLEKKIRWFIKGRKAVKKLFEKEARHLNCF | 38 | 0.26 | Anti-tubercular peptide |  |
| c_AMP_5 | RWVSKRIRKFPRKYKHILRKTILYFIGS | 28 | 0.54 | Anti-tubercular peptide |  |
| c_AMP_6 | GSSFLKGGLCGRKSGGLLQVLQRWIKG | 27 | 0.63 | Anti-tubercular peptide |  |
| c_AMP_7 | KKAARDHKRWWQVARHTARLIVGSAA | 26 | 0.93 | Anti-tubercular peptide |  |
| c_AMP_8 | REYGKRLRTGNAPLLLTGGVLALAGLLGGKRGWRRWARLALIVAPLLRRR | 49 | 1.49 | Anti-tubercular peptide |  |
| c_AMP_9 | LSRPPDVGMRWKWVLAAAAAKAALCGWHTTLLQAKTKAATLV | 42 | 0.48 | Anti-tubercular peptide |  |
| c_AMP_10 | PRWMRRFNRGMALLLLLSAWAAAFW | 25 | 1.72 | Anti-tubercular peptide | |

**Table F: THPdb2 similarity search results for lead candidate antimicrobial peptides c_AMP_1 and c_AMP_2 against the curated database of FDA-approved therapeutic peptides and proteins.** This table presents the Smith-Waterman alignment results including the peptide ID, sequence, length, best database hit, alignment length, percentage identity, similarity score, and E-value.

| **ID** | **Sequence** | **Length** | **Best THPdb2 Hit** | **Alignment Length (aa)** | **Identity (%)** | **Similarity (%)** | **E-value** |  |
| --- | --- | --- | --- | --- | --- | --- | --- | --- |
| c_AMP_1 | RWVAKRTRKFPRKYTQVAKKKTLLARLILYLIG | 33 | Th1251 | 28 | 32.1 | 78.6 | 1.7 |  |
| c_AMP_2 | GHDEAKAFMTCGLAGKRGGKAPRRWQHLGNMLNRLLSCRS | 40 | Th1388 | 12 | 66.7 | 91.7 | 6.2 | |

**Table G: *In silico* toxicity and hemolytic activity predictions of the ten shortlisted candidate antimicrobial peptides using ToxinPred 3.0 and HemoPI 2.0.** This table summarizes the predicted toxicity classification, hemolytic classification, and HC50 values for each candidate peptide.

| **ID** | **Sequence** | **ToxinPred 3.0 Prediction** | **HemoPI 2.0 Prediction** | **HC50 (μM)** |
| --- | --- | --- | --- | --- |
| c_AMP_1 | RWVAKRTRKFPRKYTQVAKKKTLLARLILYLIG | Non-Toxic | Hemolytic | 18.3 |
| c_AMP_2 | GHDEAKAFMTCGLAGKRGGKAPRRWQHLGNMLNRLLSCRS | Non-Toxic | Hemolytic | 65.6 |
| c_AMP_3 | FWLLGRWLRGLWRKRKAEQAAS | Non-Toxic | Hemolytic | 36.5 |
| c_AMP_4 | DLGIRTAEKLEKKIRWFIKGRKAVKKLFEKEARHLNCF | Non-Toxic | Hemolytic | 36.0 |
| c_AMP_5 | RWVSKRIRKFPRKYKHILRKTILYFIGS | Non-Toxic | Hemolytic | 27.1 |
| c_AMP_6 | GSSFLKGGLCGRKSGGLLQVLQRWIKG | Non-Toxic | Hemolytic | 50.4 |
| c_AMP_7 | KKAARDHKRWWQVARHTARLIVGSAA | Non-Toxic | Non-Hemolytic | 126.4 |
| c_AMP_8 | REYGKRLRTGNAPLLLTGGVLALAGLLGGKRGWRRWARLALIVAPLLRRR | Non-Toxic | Hemolytic | 28.2 |
| c_AMP_9 | LSRPPDVGMRWKWVLAAAAAKAALCGWHTTLLQAKTKAATLV | Non-Toxic | Hemolytic | 32.4 |
| c_AMP_10 | PRWMRRFNRGMALLLLLSAWAAAFW | Non-Toxic | Hemolytic | 17.3 |


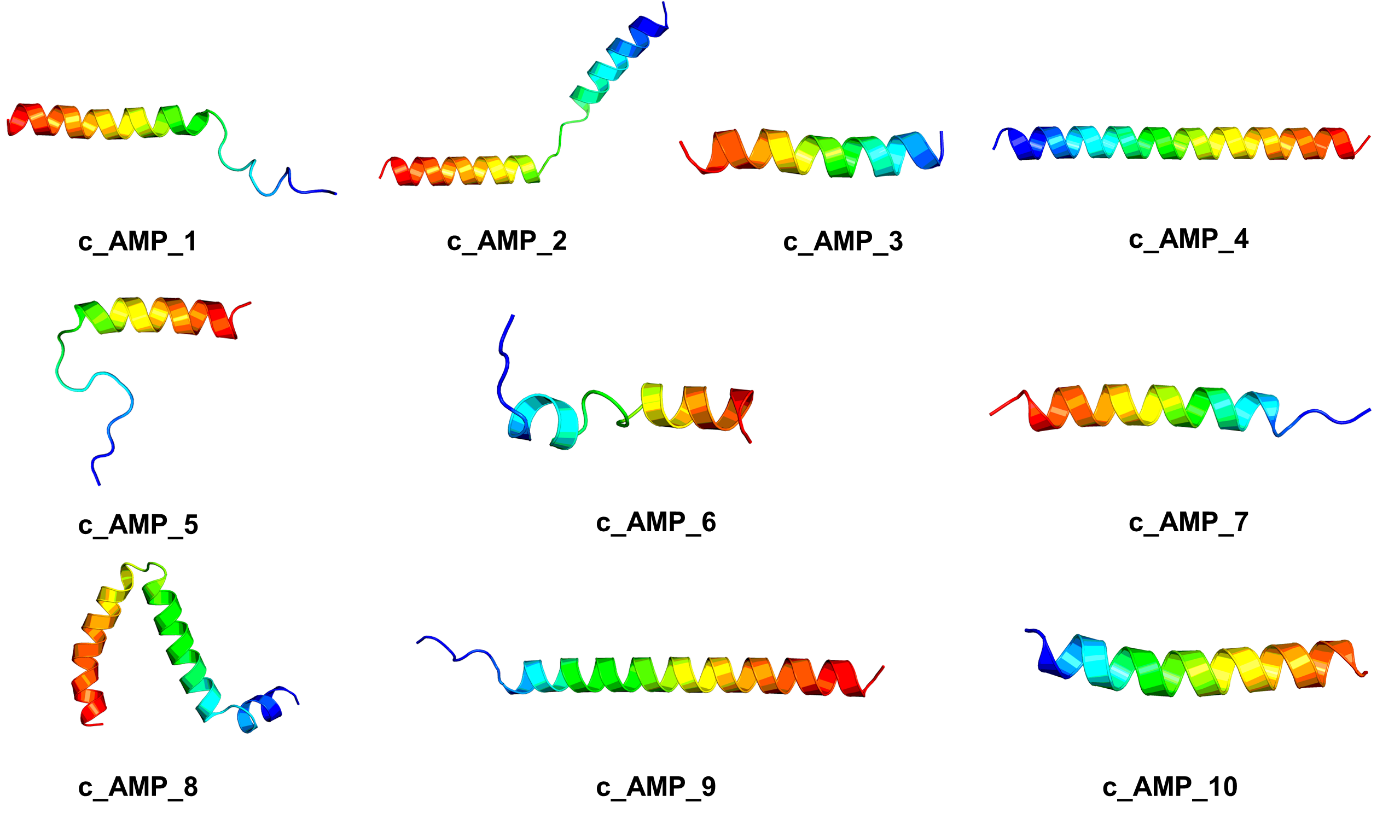


**Fig. A.** **Predicted three-dimensional structures of the top 10 cell-penetrating c_AMPs.** AlphaFold3 was used to predict the tertiary structures of the ten shortlisted antimicrobial peptides identified from Indian marine metagenomes. All peptides exhibit compact, amphipathic conformations, with several displaying distinct α-helical motifs associated with membrane interaction and cell penetration.


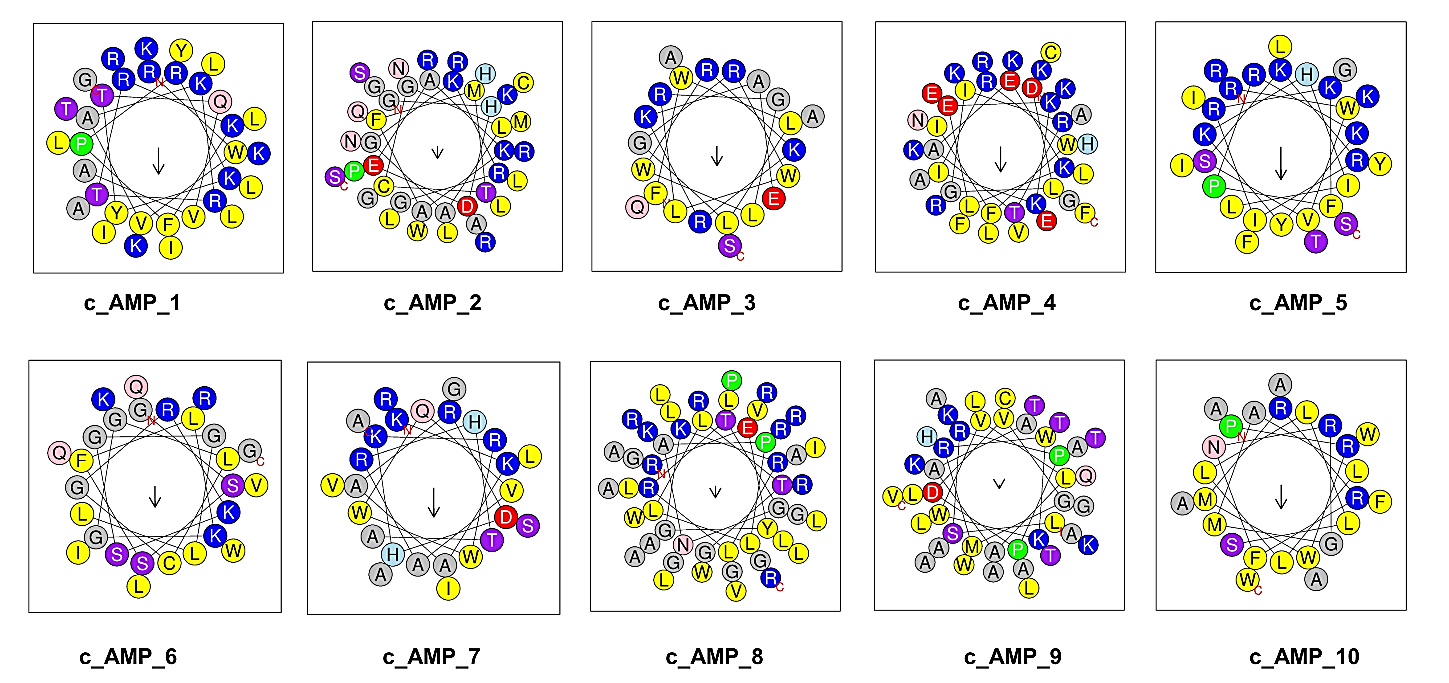


**Fig. B. Helical wheel diagrams of the 10 selected cell-penetrating c_AMPs.** Helical wheel projections illustrate the amphipathic nature of the shortlisted peptides, highlighting the spatial distribution of residues. Positively charged amino acids are shown clustered on one side (blue), while hydrophobic residues appear on the opposite face (yellow), consistent with membrane-targeting amphipathic helices.
