# Supplementary material for: In silico identification and biophysical characterization of candidate antimicrobial peptides from the Indian marine microbiome targeting multidrug-resistant ESKAPE pathogens
Source: PLoS One. 2026 Jul 22;21(7):e0353985. doi: 10.1371/journal.pone.0353985 (PMC13390849; doi:10.1371/journal.pone.0353985)
Supplement: Attachment — Submitted filename: Response to Reviewers.docx [file pone.0353985.s004.docx]

*Dear Editor,*

*We would like to express our sincere gratitude for acknowledging our manuscript and providing us the opportunity to revise it for possible publication in your esteemed journal of PLOS One.*

*We thank all the peer reviewers for their constructive and insightful suggestions towards our manuscript. Please find our detailed, point-by-point responses (****in blue italics****) to the reviewers’ comments below. We have carefully addressed each comment, conducted additional analyses where feasible, and amended the revised manuscript accordingly. We hope that our revised manuscript is now suitable for publication in the journal of “PLOS One”.*

**Point-by-point response reviewers’ comments**

**Editor**

**Comment:**

1. The manuscript in its current form overstates the experimental implications of what is fundamentally a computational study. In the current form, manuscript overstates the functional activity of the peptides; all claims should be moderated. The title and several statements in the abstract, results, and discussion should be revised to explicitly indicate that this is an in silico investigation and that the reported antimicrobial activities are predicted rather than demonstrated.

***Author’s Response***

*We sincerely thank the Editor for this important observation. We fully agree that the original manuscript language inadvertently implied experimental demonstration of antimicrobial activity. Accordingly, we have revised the title, abstract, results, and discussion to explicitly reflect the computational and predictive nature of this study. The revised title now reads: "In silico identification and biophysical characterization of candidate antimicrobial peptides from the Indian marine microbiome targeting multidrug-resistant ESKAPE pathogens." Throughout the manuscript, terms such as "dual mechanisms of disruptive action" and "functional peptide candidates" have been replaced with "distinct membrane interaction modes" and "candidate peptides," respectively, to ensure all claims are appropriately moderated and consistent with the in silico scope of the work. These changes have been made in the Title (****Page 1****), Abstract (****Page 2****), and Conclusion section (****Section 5, Page 24****) of the revised manuscript.*

**Comment:**

2. The absence of any wet-lab validation is a significant limitation and either wet-lab experiments should be done or more clearly acknowledged, accompanied by a concrete experimental roadmap (e.g., MIC testing, membrane leakage, and cytotoxicity assays).

***Author’s Response***

*We thank the Editor for raising this important point. We acknowledge that the absence of experimental validation is a significant limitation of the present study. As this work is a purely computational investigation, wet-lab experiments were beyond the scope of the current manuscript. However, we have now explicitly acknowledged this limitation and included a concrete experimental roadmap for future validation at the end of the Discussion section (****Section 4, Page 22****) of the revised manuscript. The proposed roadmap includes: (i) minimum inhibitory concentration (MIC) assays against representative ESKAPE strains, (ii) membrane permeabilization assays using fluorescent dye uptake (e.g., SYTOX Green or propidium iodide), (iii) membrane leakage assays using liposome models, and (iv) hemolysis and cytotoxicity assays on mammalian cell lines to assess selectivity and safety.*

**Comment:**

3. The rationale for selecting only two peptides for molecular dynamics simulations is insufficiently justified and should either be clarified or expanded to include additional candidates.

***Author’s Response***

*We thank the Editor for this important observation. We acknowledge that the rationale for selecting c_AMP_1 and c_AMP_2 for molecular dynamics simulations was not sufficiently articulated in the original manuscript. The manuscript already describes the upstream filtering steps, including a length cut-off of ≤50 amino acids, presence of arginine and tryptophan residues, and pLM4CPPs-based cell-penetrating peptide prediction, that reduced the candidate pool to 10 peptides (****Section 3.1, Page 12****). From these 10, c_AMP_1 and c_AMP_2 were specifically chosen for all-atom MD simulations based on a multi-criteria evaluation: (i) highest consensus scores across all six machine learning prediction tools, (ii) contrasting yet complementary physicochemical profiles - c_AMP_1 exhibiting a high net positive charge (+11) and strong amphiphilicity (1.59), while c_AMP_2 showed moderate charge (+6) and balanced hydrophobicity (0.89), (iii) structurally distinct AlphaFold3-predicted conformations, c_AMP_1 adopting a continuous α-helical structure and c_AMP_2 a semi-helical conformation with flexible termini, enabling a meaningful comparative biophysical analysis, and (iv) the considerable computational demands of 300 ns all-atom simulations across three bacterial membrane systems, which necessitated a focused selection of the most structurally divergent candidates. This rationale has now been explicitly clarified in Results (****Section 3.2, Page 13****)* *and Methods (****Section 2.4, Page 8****) of the revised manuscript.*

**Comment:**

4. Furthermore, given the reliance on Arg/Trp-based rules for cell-penetrating behavior, the authors should cross-validate their shortlisted peptides using established CPP resources such as CPPsite 2.0, CellPPDmod.

***Author’s Response***

*We thank the Editor for this valuable suggestion. As recommended, we have cross-validated all ten shortlisted peptides using CellPPD-Mod and CPPsite 3.0 (an updated version of CPPsite 2.0), in addition to the originally used pLM4CPPs model. CPP activity was independently predicted using two machine learning-based tools - pLM4CPPs and CellPPD-Mod. Nine out of ten peptides were consistently predicted as CPPs by both tools. One peptide, c_AMP_6, yielded conflicting predictions, classified as CPP by pLM4CPPs but as Non-CPP by CellPPD-Mod, and this discrepancy has been duly acknowledged in the revised manuscript. Additionally, similarity searches against the CPPsite 3.0 database of experimentally validated CPPs using the Smith-Waterman algorithm were conducted as a complementary novelty assessment. All ten candidates yielded low alignment scores (13-19) over short local windows (3-9 residues), indicating no significant full-length similarity to any known CPP in the database. The prediction thresholds applied for each tool have been clearly stated in the Methods section. These results have been incorporated into Methods (****Section 2.4, Page 8****) and Results (****Section 3.1,*** ***Page 12****) of the revised manuscript and summarized in* ***Supplementary Tables S3 and S4****.*

**Comment:**

5. In addition, many experimentally validated antitubercular peptides are absent from general AMP repositories used to train current predictors, which can affect sensitivity for Mycobacterium-active peptides. Therefore, benchmarking against specialized datasets and tools such as AntiTbPdb and AntiTbPred would also help assess the sensitivity of their pipeline, particularly for anti-mycobacterial relevance.

***Author’s Response***

*We thank the Editor for this insightful comment. We acknowledge that general AMP prediction tools may underrepresent experimentally validated antitubercular peptides in their training datasets, potentially limiting the sensitivity of the pipeline for identifying Mycobacterium-active peptides. We wish to clarify that the primary scope of the present study is the identification of candidate AMPs targeting Gram-negative ESKAPE pathogens, specifically Pseudomonas aeruginosa, Acinetobacter baumannii, and Klebsiella pneumoniae, and not Mycobacterium tuberculosis. Nevertheless, in response to the Editor's suggestion, we have conducted an exploratory screening of all ten shortlisted candidate peptides using AntiTbPred. All ten candidates were predicted as potential anti-tubercular peptides, with scores ranging from 0.26 to 1.72, suggesting possible broad-spectrum activity that warrants further investigation. We have clearly stated in the revised manuscript that this analysis is purely exploratory in nature and that any anti-mycobacterial activity requires dedicated experimental validation using specialized assays. A note acknowledging the limitation of general AMP predictors for Mycobacterium-specific activity and recommending AntiTbPdb-based benchmarking as a direction for future work has also been added. These additions have been incorporated into the Discussion section (****Section 4, Page 23****) of the revised manuscript. The AntiTbPred results are summarized in* ***Supplementary Table S5****.*

**Comment:**

6. Finally, the translational framing would be strengthened by comparing the lead peptides with characteristics of clinically approved therapeutics using curated resources such as THPdb, SATPdb etc. and by more deeply engaging with recent marine AMP and membrane-modeling literature.

***Author’s Response***

*We thank the Editor for this constructive suggestion. In response to the first part of this comment, similarity searches were performed for c_AMP_1 and c_AMP_2 against THPdb2, a curated database of FDA-approved therapeutic peptides and proteins, using the Smith-Waterman algorithm. No statistically significant matches were identified, c_AMP_1 returned a best hit with an E-value of 1.7 and c_AMP_2 with an E-value of 6.2, confirming that both lead peptides are structurally distinct from currently approved therapeutic peptides. Importantly, both candidates fall within the "Long Peptides (21-50 AA)" category of THPdb2, which encompasses 45 unique FDA-approved therapeutic peptides, indicating that their length profile is consistent with clinically approved peptide therapeutics. Furthermore, their strongly cationic nature, amphipathic architecture, and predicted membrane-targeting properties align well with the physicochemical characteristics associated with approved antimicrobial peptide therapeutics, collectively supporting their translational potential as scaffolds for future peptide-based drug development. These observations have been incorporated into the Discussion section (****Section 4, Page 23****) of the revised manuscript, and the THPdb2 similarity search results are provided in* ***Supplementary Table S6****. In response to the second part of this comment, we have expanded our engagement with recent marine AMP discovery and membrane-modeling literature by incorporating additional relevant citations in the Introduction and Discussion sections of the revised manuscript. All newly added references are highlighted in yellow in the reference section of the revised manuscript for the Editor's and Reviewer's convenience.*

**Reviewer #1**

**Comments:**

1. Dear Author, the article titled "Uncovering dual-mechanism antimicrobial peptides from the Indian marine microbiome to combat multidrug-resistant ESKAPE Pathogens" was reviewed. This article provides useful information for its readers. Please make the following correction:
Some references are outdated, please use the new references.

***Authors' Response***

*We thank the Reviewer for this comment. We have carefully reviewed all citations in the manuscript and updated or supplemented references where more recent publications are available. References pertaining to specific software tools and prediction algorithms - including MEGAHIT (Li et al., 2016), CHARMM-GUI (Lee et al., 2016), FATSLiM (Buchoux, 2017), CONAN (Mercadante et al., 2018), AMPScanner v2 (Veltri et al., 2018), and APIN (Su et al., 2019) - have been retained as their original publications, as these are the primary citations for the respective tools. For conceptual and mechanistic citations, the following older references have been supplemented with more recent literature: (i) Khandelia & Kaznessis (2007) supplemented with Necula et al. (2023) for cation-π interactions in AMP membrane binding; (ii) Kooijman et al. (2007) supplemented with Graber et al. (2022) for hydrogen bond interactions with phospholipid headgroups; (iii) Mukherjee et al. (2017) supplemented with Ma et al. (2024) for membrane thinning in MD simulations; (iv) Hong et al. (2019) supplemented with Sun et al. (2022) for melittin membrane insertion; and (v) Bobone & Stella (2019) replaced with Ma et al. (2024) for AMP selectivity toward bacterial membranes. All newly added references are highlighted in yellow in the reference section of the revised manuscript for the Editor's and Reviewer's convenience. These updates have been incorporated throughout the revised manuscript.*

**Reviewer #2**

The authors analyze five Indian marine metagenomic datasets to identify antimicrobial peptide candidates using six prediction tools. After applying additional filters based on peptide length, amino acid composition, and predicted cell-penetrating properties, they reduce the set to ten peptides. Two of these are then selected for molecular dynamics simulations with Gram-negative membrane models. The differences in how these peptides interact with the membranes are described in the Results and discussed as possible dual mechanisms. While the overall workflow is logical and clearly presented, several aspects of the methodology and interpretation require clarification and more cautious framing.

***Author’s Response***

*We sincerely thank the Reviewer for this thoughtful summary of our work and for acknowledging the logical and clear presentation of the overall workflow. We appreciate the constructive feedback regarding the areas requiring clarification and more cautious framing. We fully agree with the Reviewer's assessment that several aspects of the methodology and interpretation required additional clarity and moderation, particularly with respect to the characterization of the observed peptide-membrane interaction differences as "dual mechanisms." We have carefully addressed each of the specific concerns raised, as detailed in our point-by-point responses below. The manuscript has been revised accordingly to provide greater methodological transparency and to ensure that all interpretations are appropriately aligned with the in silico scope of the study.*

**Comment:**

1. The Methods section should clearly state (i) the total number of peptides advanced to MD simulations, (ii)Whether c_AMP_1 and c_AMP_2 were the only peptides simulated, and (iii) the criteria used to select them (Charge, hydrophobic moment, predictable activity, or structural diversity).

***Author’s Response***

*We thank the Reviewer for this important observation. We confirm that c_AMP_1 and c_AMP_2 were the only two peptides advanced to all-atom MD simulations in this study. Their selection from the ten shortlisted candidates was based on a multi-criteria evaluation framework considering: (i) net charge - c_AMP_1 exhibiting the highest net positive charge (+11) and c_AMP_2 a moderate charge (+6), representing contrasting electrostatic profiles; (ii) hydrophobic moment and amphiphilicity index - c_AMP_1 with 1.59 and c_AMP_2 with 0.89, reflecting distinct hydrophobic distributions; (iii) predicted membrane activity based on physicochemical profiling via DBAASP; and (iv) structural diversity - c_AMP_1 adopting a continuous α-helical conformation and c_AMP_2 a semi-helical structure with flexible termini as predicted by AlphaFold3. These clarifications have now been explicitly incorporated into Methods (****Section 2.4, Page 8****) and Results (****Section 3.2, Page 13****) of the revised manuscript.*

**Comment:**

2. The claim of "dual mechanisms" seems somewhat stronger than what is directly supported by the simulation results. The observed differences mainly show distinct membrane interaction behaviors in the MD simulations, rather than clearly separate antimicrobial mechanisms. The reported analyses describe interaction patterns but do not directly demonstrate effects such as pore formation or membrane permeabilization. The authors may consider softening the wording in the Discussion and Conclusions to better reflect the in silico scope of the results.

***Author’s Response***

*We thank the Reviewer for this important and valid observation. We fully agree that the MD simulation data describe distinct membrane interaction modes and orientation behaviors rather than directly demonstrating functional antimicrobial mechanisms such as pore formation or membrane permeabilization. Accordingly, we have carefully revised the Discussion and Conclusions sections to replace mechanistic terminology with language that accurately reflects the in silico nature of the observations. Specifically, references to "dual mechanisms," "toroidal pore or carpet-like disruption mechanisms," "barrel-stave or transmembrane insertion modes," and "membrane-disruptive mechanisms" have been replaced with "distinct membrane interaction modes," "surface-associated orientation consistent with carpet-like models," "deeply embedded orientation consistent with insertion-type models," and "distinct biophysical interaction profiles," respectively. These revisions ensure that the conclusions are appropriately aligned with the computational scope of the study. The changes have been made throughout the Discussion (****Section 4, Page 19-23****) and Conclusion (****Section 5, Page 24****) of the revised manuscript.*

**Comment:**

3. After the six prediction tools are applied, the candidate peptides are further filtered by length (≤ 50 amino acids) and by requiring the presence of both arginine and tryptophan residues. These additional filters narrow the set toward cationic, membrane-active peptides and likely exclude other AMP types. It would be helpful if the authors briefly note this limitation in the Discussion.

***Author’s Response***

*We thank the Reviewer for this valid observation. We agree that the application of length and Arg/Trp compositional filters inherently narrows the candidate set toward cationic, membrane-active peptides and may exclude other biologically relevant AMP types, such as anionic peptides, proline-rich AMPs, or peptides acting through intracellular mechanisms. This is an important limitation of the current pipeline and has now been explicitly acknowledged in the Discussion section (****Section 4, Page 19****) of the revised manuscript.*

**Comment:**

4. The manuscript states that agreement across six prediction tools produces a high-confidence AMP set, but no quantitative validation or benchmarking results are shown to support this claim (for example, testing on known AMP and non-AMP reference datasets). The authors should either provide validation metrics or soften this statement. In addition, a few MD simulation details could be described more clearly to support reproducibility.

***Author’s Response***

*We thank the Reviewer for this critical observation. We acknowledge that the claim of a "high-confidence" AMP set based solely on consensus across six prediction tools was not supported by quantitative validation metrics in the original manuscript. We wish to clarify that each of the six tools used - AMPlify, ampir, AMPScanner v2, AI4AMP, amPEPpy, and APIN - has been independently validated and benchmarked against curated AMP and non-AMP reference datasets in their respective original publications, with reported accuracy values ranging from 85% to 97%. The use of a consensus-based approach requiring agreement across all six independently validated tools further reduces the likelihood of false positives, as peptides predicted by all six diverse algorithmic frameworks are more likely to represent true AMPs. However, we acknowledge that no additional independent benchmarking was performed within this study. Accordingly, we have softened the language in the Methods and Discussion sections to more accurately reflect this, replacing "ensuring a high-confidence set" with "suggesting a high-confidence set" and adding a clarifying statement that the confidence claim is supported by the independent validation of each constituent tool rather than by additional in-study benchmarking. These changes have been incorporated into Methods (****Section 2.3, Page 7****) and the Discussion section (****Section 4, Page 18-19****) of the revised manuscript.*

**Comment:**

5. For reproducibility, a few MD setup details should be clarified. Please specify the initial peptide placement and orientation relative to the membrane, whether simulations were run with replicates or single trajectories, and the criteria used to define peptide-lipid hydrogen bonds. A brief note on possible timescale limitations for the membrane simulations, especially LPS-containing models, would also help interpretation.

***Author’s Response***

*We thank the Reviewer for these important observations regarding reproducibility. We have addressed each point specifically as follows. Regarding initial peptide placement and orientation, both were handled using the default parameters of CHARMM-GUI during system assembly, which automatically positions the peptide above the membrane surface in the aqueous phase at an appropriate distance from the lipid headgroup layer. Regarding simulation replicates, we acknowledge that each system was run as a single trajectory due to the considerable computational demands of six independent all-atom 300 ns simulations, and this has now been explicitly noted as a limitation in the revised manuscript. Regarding hydrogen bond criteria, we have clarified in the Methods section that hydrogen bonds were defined using a donor-acceptor distance cutoff of ≤3.0 Å and a donor-hydrogen-acceptor angle of ≥150°, consistent with standard GROMACS hydrogen bond definitions. Regarding timescale limitations, we have added a brief note in the Discussion acknowledging that 300 ns simulations, while extensive for single-peptide systems, may not fully capture slow membrane reorganization events, particularly in the LPS-containing A. baumannii outer membrane model where lipid dynamics operate on longer timescales. These clarifications have been incorporated into Methods (****Section 2.5, Page 9****), Methods (****Section 2.6, Page 10****), Methods (****Section 2.7, Page 11****), and the Discussion section (****Section 4, Page 22-23****) of the revised manuscript.*

**Comment:**

6. The paper presents the results as if they directly apply to treating multidrug-resistant ESKAPE pathogens and suggests therapeutic potential in the Title, Abstract, and Conclusions. However, the study is entirely computational and does not include experimental validation. The wording should be revised to clearly reflect that the results are based only on in-silico analysis.

***Author’s Response***

*We thank the Reviewer for this important observation. We agree that several statements across the manuscript inadvertently implied direct experimental applicability. We have carefully reviewed the entire manuscript and moderated all such language to accurately reflect the in silico scope of this study. Specifically, terms such as "confirms," "validates," "demonstrates," and "establishes" have been replaced with "suggests," "indicates," or "predicts" where appropriate, and phrases implying direct therapeutic applicability have been qualified with "pending experimental validation" throughout. These changes have been incorporated across the Results (****Section 3.3, Page 14; Section 3.4, Page 15; Section 3.5, Page 16; Section 3.6, Page 17****) and Conclusion (****Section 5, Page 24****) sections of the revised manuscript.*

**Reviewer #3**

The manuscript repeatedly frames the observed behaviors of c_AMP_1 and c_AMP_2 as evidence of distinct “dual antimicrobial mechanisms.” While the molecular dynamics simulations are extensive (300 ns) and technically well executed, this interpretation exceeds what can be robustly inferred from single-peptide, in silico membrane simulations.
Specifically, the two behaviors described—(i) surface-associated, parallel alignment of c_AMP_1 and (ii) deeper, tilted or membrane-inserted orientation of c_AMP_2—represent distinct membrane interaction modes, not demonstrated antimicrobial mechanisms. MD simulations of individual peptides can reveal geometric preferences, stability, and lipid perturbation patterns, but they do not, in isolation, establish functional mechanisms such as carpet-like disruption, pore formation, or bactericidal activity.
At several points in the manuscript (title, abstract, Results, and Discussion), language implies functional antimicrobial mechanisms (e.g., “dual-mechanism AMPs,” “disruptive action,” “therapeutic potential”), which are not experimentally validated and cannot be conclusively supported by MD data alone. This framing risks overstating the biological implications of the simulations and conflating compatibility with known models with demonstration of mechanism.

***Author’s Response***

*We sincerely thank the Reviewer for this thorough and perceptive evaluation. We fully accept this critique and acknowledge that the original framing of the manuscript inadvertently conflated compatibility with known membrane interaction models with demonstration of functional antimicrobial mechanisms. We agree that the observed behaviors of c_AMP_1 and c_AMP_2, specifically the surface-associated parallel alignment of c_AMP_1 and the deeper, tilted membrane-inserted orientation of c_AMP_2, represent distinct membrane interaction modes inferred from in silico simulations, and that these observations cannot, in isolation, establish functional outcomes such as pore formation, carpet-like disruption, or bactericidal activity. We have undertaken a careful and systematic revision of the manuscript's interpretative framework to address this concern, replacing all mechanistic terminology with language accurately describing predicted interaction modes, orientation states, and membrane perturbation patterns. The study has been explicitly reframed as a computational prioritization and biophysical characterization of candidate AMPs throughout the title, abstract, results, discussion, and conclusions. The specific revisions made in response to each of the Reviewer's individual points are addressed below.*

To strengthen the manuscript and align conclusions with the presented evidence, the authors should:

**Comment:**

1. Reframe the study explicitly as a computational prioritization and biophysical characterization of candidate AMPs, rather than as a demonstration of antimicrobial mechanisms.

***Author’s Response***

*We thank the Reviewer for this important and valid suggestion. We fully agree that the original framing of the study inadvertently implied a demonstration of antimicrobial mechanisms rather than a computational prioritization and biophysical characterization of candidate AMPs. The study has now been explicitly reframed throughout the manuscript to reflect its true scope - an in silico pipeline for the identification, prioritization, and biophysical characterization of candidate AMPs from Indian marine metagenomic datasets. This reframing has been applied consistently across the title, abstract, introduction, results, discussion, and conclusions. These revisions have been incorporated into the Introduction (****Section 1, Page 6****) of the revised manuscript.*

**Comment:**

2. Replace mechanistic terminology (e.g., “dual mechanism,” “pore-forming,” “disruptive action”) with language describing interaction modes, orientation states, or membrane perturbation patterns inferred from simulations.

***Author’s Response***

*We thank the Reviewer for this specific and constructive suggestion. We have systematically replaced all mechanistic terminology throughout the manuscript with language that accurately describes the observed interaction modes, orientation states, and membrane perturbation patterns inferred from the MD simulations. These changes have been incorporated across the Abstract (****Page 1****), Discussion (****Section 4, Page 19-23****), and Conclusion (****Section 5, page 24****) of the revised manuscript.*

**Comment:**

3. Clearly state the limitations of single-peptide MD simulations and avoid implying bactericidal function or therapeutic efficacy in the absence of experimental validation.

***Author’s Response***

*We thank the Reviewer for this important point. We fully agree that single-peptide MD simulations have inherent limitations that must be clearly acknowledged. While these simulations provide valuable insights into geometric preferences, orientation states, and lipid perturbation patterns of individual peptides, they cannot, in isolation, establish functional outcomes such as bactericidal activity, pore formation, or therapeutic efficacy. Accordingly, we have added a dedicated paragraph in the Discussion section explicitly acknowledging these limitations. Specifically, we note that: (i) single-peptide simulations do not capture the cooperative behavior of multiple peptides that is often required for membrane disruption; (ii) each simulation was run as a single trajectory, which limits statistical confidence; (iii) the 300 ns timescale, while extensive for single-peptide systems, may not fully capture slow membrane reorganization events, particularly in the LPS-containing asymmetric outer membrane model of A. baumannii; and (iv) the absence of experimental validation means that all observations remain predictive rather than demonstrated. All language implying bactericidal function or therapeutic efficacy has been moderated accordingly throughout the manuscript. These additions have been incorporated into the Discussion section (****Section 4, Page 22-23****) of the revised manuscript.*

**Comment:**

4. Ensure consistency of this revised framing across the title, abstract, Results, Discussion, and Conclusions.

***Author’s Response***

*We thank the Reviewer for this important point. We have conducted a thorough review of the entire manuscript to ensure that the revised computational framing is applied consistently across all sections. The title, abstract, introduction, results, discussion, and conclusions have all been carefully checked and moderated to ensure that no residual mechanistic or overstated language remains. These revisions have been incorporated throughout the revised manuscript.*

**Comment:**

5. Addressing this conceptual issue does not require additional experimental data, but it does require a careful and systematic revision of the manuscript’s interpretative framework.

***Author’s Response***

*We thank the Reviewer for this clarification. We acknowledge and appreciate the Reviewer's recognition that the conceptual concerns raised can be addressed through a careful revision of the manuscript's interpretative framework rather than through additional experimental work. As detailed in our responses to comments 1-4, we have undertaken a systematic and thorough revision of the manuscript's interpretative framing across all sections. The study has been explicitly reframed as a computational prioritization and biophysical characterization of candidate AMPs, all mechanistic terminology has been replaced with language describing interaction modes and orientation states inferred from simulations, the limitations of single-peptide MD simulations have been clearly stated, and consistency of this revised framing has been ensured across the title, abstract, results, discussion, and conclusions. We trust that these comprehensive revisions adequately address the Reviewer's concerns.*

**Comment:**

6. The membrane models used for *A. baumannii*, *K. pneumoniae*, and *P. aeruginosa* are not equivalent. While *A. baumannii* is modeled using an asymmetric outer membrane containing Lipid A/LPS, *K. pneumoniae* and *P. aeruginosa* are represented by symmetric phospholipid bilayers lacking LPS. This distinction is not clearly justified or discussed, yet results are directly compared across systems. Given the known impact of LPS on peptide binding, insertion, and membrane perturbation, the authors should explicitly clarify the rationale for using different membrane architectures and discuss the implications and limitations this imposes on cross-species comparisons.

***Author’s Response***

*We thank the Reviewer for this critical and highly valid observation. We fully acknowledge that the three membrane models employed in this study are not architecturally equivalent, an asymmetric outer membrane model containing Lipid A/LPS was used for A. baumannii, while symmetric phospholipid bilayers lacking LPS were used for K. pneumoniae and P. aeruginosa. The rationale for this distinction is rooted in the biological reality and availability of validated lipidomic data for each organism. For A. baumannii, well-characterized species-specific lipidomic studies and computational membrane models incorporating the asymmetric LPS-containing outer membrane are available and have been previously validated (Jiang et al., 2020), making it the most biologically representative model for this organism. For K. pneumoniae and P. aeruginosa, validated inner membrane lipidomic compositions were available and employed based on aggregated lipidomic data (Gazerani et al., 2025), whereas equivalent outer membrane models incorporating species-specific LPS compositions for these organisms were not available at the time of this study. We acknowledge that this architectural difference has important implications for cross-species comparisons - LPS is known to significantly influence peptide binding, insertion depth, and membrane perturbation patterns through its bulky hydrophilic polysaccharide chains and strong electrostatic interactions. Therefore, the observed differences in peptide behavior across the three membrane systems reflect not only species-specific lipid compositions but also the fundamentally different membrane architectures modeled, and direct quantitative comparisons across systems should be interpreted with caution. This rationale and the associated limitations have now been explicitly stated in Methods (****Section 2.5, Page 9****) and the Discussion section (****Section 4, Page 21****) of the revised manuscript.*

**Comment:**

7. The in silico toxicity and hemolysis predictors suggest a favorable safety profile; the authors could include this in silico profile.

***Author’s Response***

*We thank the Reviewer for this valuable suggestion. In response, we have performed in silico toxicity and hemolytic activity predictions for all ten shortlisted candidate peptides using ToxinPred 3.0 and HemoPI 2.0 respectively. ToxinPred 3.0 predicted all ten peptides as non-toxic, suggesting a favorable mammalian safety profile. HemoPI 2.0 predicted nine out of ten peptides as hemolytic based on binary classification; however, the predicted HC50 values - the concentration required to lyse 50% of red blood cells - ranged from 18.3 μM to 126.4 μM, with c_AMP_2 showing a notably higher HC50 of 65.6 μM and c_AMP_7 classified as non-hemolytic with an HC50 of 126.4 μM. These HC50 values indicate variable but moderate predicted hemolytic potency, and it should be noted that in silico hemolysis predictions are inherently limited in accuracy and that experimental hemolysis assays on mammalian red blood cells remain essential for definitive safety assessment. These results have been incorporated into Methods (****Section 2.4, Page 8****), Results (****Section 3.1, Page 12****), and the Discussion section (****Section 4, Page 19****) of the revised manuscript and summarized in* ***Supplementary Table S7****.*
