# Supplementary material for: In silico identification and biophysical characterization of candidate antimicrobial peptides from the Indian marine microbiome targeting multidrug-resistant ESKAPE pathogens
Source: PLoS One. 2026 Jul 22;21(7):e0353985. doi: 10.1371/journal.pone.0353985 (PMC13390849; doi:10.1371/journal.pone.0353985)
Supplement: Attachment — Submitted filename: Response_to_Reviewers_auresp_2.docx [file pone.0353985.s005.docx]

*Dear Editor,*

*We would like to express our sincere gratitude for acknowledging our manuscript and providing us the opportunity to revise it for possible publication in your esteemed journal of PLOS One.*

*We thank all the peer reviewers for their constructive and insightful suggestions towards our manuscript. Please find our detailed, point-by-point responses (****in blue italics****) to the reviewers’ comments below. We have carefully addressed each comment, conducted additional analyses where feasible, and amended the revised manuscript accordingly. We hope that our revised manuscript is now suitable for publication in the journal of “PLOS One”.*

**Point-by-point response reviewers’ comments**

**Reviewer #2**

The authors have satisfactorily addressed the concerns raised in the previous round of review. The manuscript has improved in terms of clarity, methodological description, and overall framing. In particular, the interpretation of the molecular dynamics results has been revised appropriately, and the conclusions are now better aligned with the in-silico nature of the study. The rationale for peptide selection and the description of the simulation setup is now clearer. The manuscript also includes a more explicit discussion of its limitations, including the absence of experimental validation and independent benchmarking.

***Author’s Response***

*We sincerely thank the Reviewer for this positive and encouraging assessment of the revised manuscript. We are glad that the revisions addressing the interpretation of molecular dynamics results, the clarity of the peptide selection rationale, the simulation setup description, and the explicit discussion of limitations have met the Reviewer's expectations. We have further addressed the single remaining minor point identified below.*

**Comment:**

1. One minor point remains regarding consistency of wording. In a few sections, particularly the conclusion, the language still slightly overemphasizes potential therapeutic implications. This should be moderated to ensure it remains fully consistent with the computational scope of the study.

***Author’s Response***

*We thank the Reviewer for this observation. We agree that the language in a few sections inadvertently overemphasized the therapeutic implications of the study beyond what is directly supported by the in silico evidence. Accordingly, we have carefully reviewed the manuscript and made targeted revisions in the Abstract (****Page 2****), Introduction (****Section 1, Page 6****), Discussion (****Section 4, Page 23****) and Conclusion (****Section 5, Page 24****) to moderate such language. Phrases implying confirmed findings or direct therapeutic applicability have been replaced with appropriately qualified expressions that accurately reflect the computational and predictive scope of the study. We believe these revisions ensure that the manuscript is now fully consistent in its framing throughout.*

**Reviewer #3**

No comments were provided.

***Author’s Response***

*We thank the reviewer for recommending our work for publication.*
