# Supplementary material for: In silico identification and biophysical characterization of candidate antimicrobial peptides from the Indian marine microbiome targeting multidrug-resistant ESKAPE pathogens
Source: PLoS One. 2026 Jul 22;21(7):e0353985. doi: 10.1371/journal.pone.0353985 (PMC13390849; doi:10.1371/journal.pone.0353985)
Supplement: Attachment — Submitted filename: Response_to_Reviewers_auresp_3.docx [file pone.0353985.s006.docx]

*Dear Editor,*

*We would like to express our sincere gratitude for acknowledging our manuscript and providing us the opportunity to revise it for possible publication in your esteemed journal of PLOS One.*

*We thank all the peer reviewers for their constructive and insightful suggestions towards our manuscript. Please find our detailed, point-by-point responses (****in blue italics****) to the reviewer’s comments below. We have carefully addressed each comment* *and amended the revised manuscript accordingly, with all changes marked using track changes. We hope that our revised manuscript is now suitable for publication in the journal of “PLOS One”.*

**Point-by-point response reviewers’ comments**

**Reviewer #2**

The revised manuscript is improved compared to the previous version, and the authors have addressed many of the earlier concerns. The topic is important, and the overall workflow of the
study is presented clearly.

***Author’s Response***

*We sincerely thank the Reviewer for the careful re-evaluation of our manuscript and for acknowledging the improvements made in the revised version. We are grateful for the recognition that the topic is important and that the overall workflow is presented clearly. We have taken the remaining comments seriously and have made targeted revisions throughout the Abstract, Results, Discussion, and Conclusion to ensure that the language is fully consistent with the computational and predictive nature of the study, that the membrane-interaction results are interpreted cautiously in the absence of experimental validation, and that the number of peptides used for the molecular dynamics simulations is stated unambiguously. Our point-by-point responses are provided below, and all corresponding changes have been marked using track changes in the revised manuscript.*

**Comment:**

1. Some parts of the manuscript still sound stronger than what is shown in the study. The MD simulations show different ways the peptides interact with the membranes, but they do not directly prove antimicrobial mechanisms or therapeutic effects.

***Author’s Response***

*We thank the Reviewer for this important observation. We fully agree that the molecular dynamics simulations characterize predicted peptide-membrane interaction modes and do not, in themselves, demonstrate antimicrobial mechanisms or therapeutic effects. Accordingly, we have revised the concluding statements of the Abstract (****Page 2****), the Discussion (****Section 4, Page 23****), and the Conclusion (****Section 5,*** ***Page 24****) to remove wording that could imply proven activity or therapeutic readiness. Expressions such as “effectively prioritizes,” “conducive to therapeutic development,” and “strongly support” have been replaced with appropriately qualified phrasing that frames the peptides as computational candidates requiring experimental validation. We note that the manuscript already contains a dedicated paragraph in the Discussion explicitly stating that single-peptide MD simulations cannot establish pore formation, permeabilization, or bactericidal activity; the present revisions bring the surrounding summary statements into line with that caveat.*

**Comment:**

2. The wording in the Abstract, Results, Discussion and Conclusion should be made more careful and consistent with the computational nature of the work.

***Author’s Response***

*We thank the Reviewer for this suggestion and have carried out a careful pass across the Abstract, Results, Discussion, and Conclusion to harmonize the language with the computational scope of the work. Subjective or comparative terms that implied functional superiority, such as “underperformed” and “superior membrane adaptability” in Results (****Section 3.5, Page 17****), have been rephrased in neutral, observation-based terms tied explicitly to the simulated systems. Verbs describing simulation outcomes such as “revealed,” “exhibited” have, where appropriate, been softened to “indicated” or “displayed,” and qualifiers such as “in the simulations” / “in silico” have been retained or added so that descriptive and interpretive statements read consistently throughout. Together with the changes made under the preceding and following comments, these edits ensure a uniform, computationally qualified tone across all four sections.*

**Comment:**

3. There are still a few inconsistencies regarding the peptides used for the MD simulations. In some sections, three peptides are mentioned, while the detailed analysis is only presented for c_AMP_1 and c_AMP_2. This should be corrected throughout the manuscript.

***Author’s Response***

*We thank the Reviewer for highlighting this point and apologize for the lack of clarity. We confirm that exactly two peptides, c_AMP_1 and c_AMP_2, were advanced to molecular dynamics simulations; all detailed analyses correspond to these two peptides. The recurring reference to “three” in the manuscript denotes the three bacterial membrane systems (A. baumannii, K. pneumoniae, and P. aeruginosa), not three peptides, and the close juxtaposition of “two peptides” and “three membranes” may have caused this ambiguity. To remove any possible confusion, we have revised the simulation-design statement in Materials and methods (****Section 2.5, Page 9****) to state the design explicitly as “two peptides × three membranes = six independent MD systems,” and we have re-checked the Abstract, Methods (****Sections 2.4-2.5****), Results (****Sections 3.3-3.6****), and Discussion (****Section 4****) to ensure that the two-peptide selection and the three-membrane design are stated consistently and cannot be conflated. The peptide count is now unambiguous throughout.*

**Comment:**

4. In some places, the interpretation of the membrane interaction results goes beyond what can be directly concluded from the simulations. Since no experimental validation has been included, these points should be discussed more cautiously.

***Author’s Response***

*We agree with the Reviewer that, in the absence of experimental validation, certain interpretations were stated more definitively than the simulations permit. We have therefore revised these statements to distinguish what is directly observed in the trajectories from what would require experimental confirmation. In Results (****Section 3.5, Page 17****), the causal phrasing “indicating that c_AMP_2 inserts more deeply… and induces localized lipid compression” has been changed to the observation-level “consistent with deeper insertion… within the simulated bilayers.” In the Discussion (****Section 4, Page 21****), the inference that a deeper orientation “could enhance permeabilization” has been qualified to state explicitly that permeabilization cannot be inferred from structural simulations alone and requires dedicated experimental assays. These revisions complement the existing limitations paragraph in the Discussion, which already notes that single-peptide simulations cannot establish pore formation or bactericidal activity, and ensure that all mechanistic interpretations are now framed as predicted, simulation-derived observations.*
