# Supplementary material for: In silico identification and biophysical characterization of candidate antimicrobial peptides from the Indian marine microbiome targeting multidrug-resistant ESKAPE pathogens
Source: PLoS One. 2026 Jul 22;21(7):e0353985. doi: 10.1371/journal.pone.0353985 (PMC13390849; doi:10.1371/journal.pone.0353985)
Supplement: Peer review history

# Peer review: Decision Letter 0

**DOI:** 10.1371/journal.pone.0353985.r001

**Authors:** Salman Sadullah Usmani

10 Feb 2026

PONE-D-25-55465

Uncovering dual-mechanism antimicrobial peptides from the Indian marine microbiome to combat multidrug-resistant ESKAPE Pathogens

PLOS One

Dear Dr. Dehury,

Thank you for submitting your manuscript to PLOS ONE. After careful consideration, we feel that it has merit but does not fully meet PLOS ONE’s publication criteria as it currently stands. Therefore, we invite you to submit a revised version of the manuscript that addresses the points raised during the review process.

Please submit your revised manuscript by Mar 27 2026 11:59PM. If you will need more time than this to complete your revisions, please reply to this message or contact the journal office at plosone@plos.org. When you're ready to submit your revision, log on to https://www.editorialmanager.com/pone/ and select the 'Submissions Needing Revision' folder to locate your manuscript file.

Please include the following items when submitting your revised manuscript:

- A letter that responds to each point raised by the academic editor and reviewer(s). You should upload this letter as a separate file labeled 'Response to Reviewers'.
- A marked-up copy of your manuscript that highlights changes made to the original version. You should upload this as a separate file labeled 'Revised Manuscript with Track Changes'.
- An unmarked version of your revised paper without tracked changes. You should upload this as a separate file labeled 'Manuscript'.

If you would like to make changes to your financial disclosure, please include your updated statement in your cover letter. Guidelines for resubmitting your figure files are available below the reviewer comments at the end of this letter.

If applicable, we recommend that you deposit your laboratory protocols in protocols.io to enhance the reproducibility of your results. Protocols.io assigns your protocol its own identifier (DOI) so that it can be cited independently in the future. For instructions see: https://journals.plos.org/plosone/s/submission-guidelines#loc-laboratory-protocols. Additionally, PLOS ONE offers an option for publishing peer-reviewed Lab Protocol articles, which describe protocols hosted on protocols.io. Read more information on sharing protocols at https://plos.org/protocols?utm_medium=editorial-email&utm_source=authorletters&utm_campaign=protocols.

We look forward to receiving your revised manuscript.

Kind regards,

Salman Sadullah Usmani, Ph.D.

Academic Editor

PLOS One

**Journal Requirements:**

1. When submitting your revision, we need you to address these additional requirements.

Please ensure that your manuscript meets PLOS ONE's style requirements, including those for file naming. The PLOS ONE style templates can be found at

https://journals.plos.org/plosone/s/file?id=wjVg/PLOSOne_formatting_sample_main_body.pdf and

https://journals.plos.org/plosone/s/file?id=ba62/PLOSOne_formatting_sample_title_authors_affiliations.pdf

2. Please note that PLOS One has specific guidelines on code sharing for submissions in which author-generated code underpins the findings in the manuscript. In these cases, we expect all author-generated code to be made available without restrictions upon publication of the work. Please review our guidelines at https://journals.plos.org/plosone/s/materials-and-software-sharing#loc-sharing-code and ensure that your code is shared in a way that follows best practice and facilitates reproducibility and reuse.

3. Thank you for stating the following in the Acknowledgments Section of your manuscript:

The authors would like to thank Manipal School of Life Sciences, Manipal Academy of Higher Education for providing necessary facilities to carry out this work. The authors also acknowledge the high-performance computing facility and storage provided by CDAC-MAHE, Param Utkarsh facility. The authors also acknowledge the funding Anusandhan National Research Foundation (Sanction Order: ANRF/ECRG/2024/003501/LS) for providing Prime Minister Early Career Research Grant to BD.

We note that you have provided funding information that is not currently declared in your Funding Statement. However, funding information should not appear in the Acknowledgments section or other areas of your manuscript. We will only publish funding information present in the Funding Statement section of the online submission form.

Please remove any funding-related text from the manuscript and let us know how you would like to update your Funding Statement. Currently, your Funding Statement reads as follows:

The author(s) received no specific funding for this work.

Please include your amended statements within your cover letter; we will change the online submission form on your behalf.

4. If the reviewer comments include a recommendation to cite specific previously published works, please review and evaluate these publications to determine whether they are relevant and should be cited. There is no requirement to cite these works unless the editor has indicated otherwise.

**Additional Editor Comments:**

The manuscript in its current form overstates the experimental implications of what is fundamentally a computational study. In the current form, manuscript overstates the functional activity of the peptides; all claims should be moderated. The title and several statements in the abstract, results, and discussion should be revised to explicitly indicate that this is an in silico investigation and that the reported antimicrobial activities are predicted rather than demonstrated. The absence of any wet-lab validation is a significant limitation and either wet-lab experiments should be done or more clearly acknowledged, accompanied by a concrete experimental roadmap (e.g., MIC testing, membrane leakage, and cytotoxicity assays). The rationale for selecting only two peptides for molecular dynamics simulations is insufficiently justified and should either be clarified or expanded to include additional candidates. Furthermore, given the reliance on Arg/Trp-based rules for cell-penetrating behavior, the authors should cross-validate their shortlisted peptides using established CPP resources such as CPPsite 2.0, CellPPDmod. In addition, many experimentally validated antitubercular peptides are absent from general AMP repositories used to train current predictors, which can affect sensitivity for Mycobacterium-active peptides. Therefore, benchmarking against specialized datasets and tools such as AntiTbPdb and AntiTbPred would also help assess the sensitivity of their pipeline, particularly for anti-mycobacterial relevance. Finally, the translational framing would be strengthened by comparing the lead peptides with characteristics of clinically approved therapeutics using curated resources such as THPdb, SATPdb etc. and by more deeply engaging with recent marine AMP and membrane-modeling literature.

[Note: HTML markup is below. Please do not edit.]

Reviewers' comments:

Reviewer's Responses to Questions

**Comments to the Author**

1. Is the manuscript technically sound, and do the data support the conclusions?

The manuscript must describe a technically sound piece of scientific research with data that supports the conclusions. Experiments must have been conducted rigorously, with appropriate controls, replication, and sample sizes. The conclusions must be drawn appropriately based on the data presented.

Reviewer #1: Yes

Reviewer #2: Partly

Reviewer #3: Partly

**********

2. Has the statistical analysis been performed appropriately and rigorously?

Reviewer #1: Yes

Reviewer #2: N/A

Reviewer #3: No

**********

3. Have the authors made all data underlying the findings in their manuscript fully available?

The PLOS Data policy requires authors to make all data underlying the findings described in their manuscript fully available without restriction, with rare exception (please refer to the Data Availability Statement in the manuscript PDF file). The data should be provided as part of the manuscript or its supporting information, or deposited to a public repository. For example, in addition to summary statistics, the data points behind means, medians and variance measures should be available. If there are restrictions on publicly sharing data—e.g. participant privacy or use of data from a third party—those must be specified.

Reviewer #1: Yes

Reviewer #2: Yes

Reviewer #3: Yes

**********

4. Is the manuscript presented in an intelligible fashion and written in standard English?

PLOS ONE does not copyedit accepted manuscripts, so the language in submitted articles must be clear, correct, and unambiguous. Any typographical or grammatical errors should be corrected at revision, so please note any specific errors here.

Reviewer #1: Yes

Reviewer #2: Yes

Reviewer #3: No

**********

5. Review Comments to the Author

Please use the space provided to explain your answers to the questions above. You may also include additional comments for the author, including concerns about dual publication, research ethics, or publication ethics. (Please upload your review as an attachment if it exceeds 20,000 characters)

**Reviewer #1:** Dear Author,

The article titled "Uncovering dual-mechanism antimicrobial peptides from the Indian marine microbiome

to combat multidrug-resistant ESKAPE Pathogens" was reviewed. This article provides useful information for its readers. Please make the following correction:

Some references are outdated, please use the new references.

Kind regards

**Reviewer #2:** The authors analyze five Indian marine metagenomic datasets to identify antimicrobial peptide candidates using six prediction tools. After applying additional filters based on peptide length, amino acid composition, and predicted cell-penetrating properties, they reduce the set to ten peptides. Two of these are then selected for molecular dynamics simulations with Gram-negative membrane models. The differences in how these peptides interact with the membranes are described in the Results and discussed as possible dual mechanisms. While the overall workflow is logical and clearly presented, several aspects of the methadology and interpretation require clarification and more cautious framing.

The Methods section should clearly state (i) the total number of peptides advanced to MD simulations, (ii)Whether c_AMP_1 and c_AMP_2 were the only peptides simulated, and (iii) the criteria used to select them ( Charge, hydrophobic moment, predictable activity, or structural diversity).

The claim of "dual mechanisms" seems somewhat stronger than what is directly supported by the simulation results. The observed differences mainly show distinct membrane interaction behaviors in the MD simulations, rather than clearly separate antimicrobial mechanisms. The reported analyses describe interaction patterns but do not directly demonstrate effects such as pore formation or membrane permeabilization. The authors may consider softening the wording in the Discussion and Conclusions to better reflect the in silico scope of the results.

After the six prediction tools are applied, the candidate peptides are further filtered by length (≤ 50 amino acids) and by requiring the presence of both arginine and tryptophan residues. These additional filters narrow the set toward cationic, membrane-active peptides and likely exclude other AMP types. It would be helpful if the authors briefly note this limitation in the Discussion.

The manuscript states that agreement across six prediction tools produces a high-confidence AMP set, but no quantitative validation or benchmarking results are shown to support this claim (for example, testing on known AMP and non-AMP reference datasets). The authors should either provide validation metrics or soften this statement. In addition, a few MD simulation details could be described more clearly to support reproducibility.

For reproducibility, a few MD setup details should be clarified. Please specify the initial peptide placement and orientation relative to the membrane, whether simulations were run with replicates or single trajectories, and the criteria used to define peptide-lipid hydrogen bonds. A brief note on possible timescale limitations for the membrane simulations, especially LPS-containing models, would also help interpretation.

The paper presents the results as if they directly apply to treating multidrug-resistant ESKAPE pathogens and suggests therapeutic potential in the Title, Abstract, and Conclusions. However, the study is entirely computational and does not include experimental validation. The wording should be revised to clearly reflect that the results are based only on in-silico analysis.

Recommendation: Major revision. The study is interesting and technically solid, but several clarifications and wording revisions are needed before it is suitable for publication.

**Reviewer #3:** The manuscript repeatedly frames the observed behaviors of c_AMP_1 and c_AMP_2 as evidence of distinct “dual antimicrobial mechanisms.” While the molecular dynamics simulations are extensive (300 ns) and technically well executed, this interpretation exceeds what can be robustly inferred from single-peptide, in silico membrane simulations.

Specifically, the two behaviors described—(i) surface-associated, parallel alignment of c_AMP_1 and (ii) deeper, tilted or membrane-inserted orientation of c_AMP_2—represent distinct membrane interaction modes, not demonstrated antimicrobial mechanisms. MD simulations of individual peptides can reveal geometric preferences, stability, and lipid perturbation patterns, but they do not, in isolation, establish functional mechanisms such as carpet-like disruption, pore formation, or bactericidal activity.

At several points in the manuscript (title, abstract, Results, and Discussion), language implies functional antimicrobial mechanisms (e.g., “dual-mechanism AMPs,” “disruptive action,” “therapeutic potential”), which are not experimentally validated and cannot be conclusively supported by MD data alone. This framing risks overstating the biological implications of the simulations and conflating compatibility with known models with demonstration of mechanism.

To strengthen the manuscript and align conclusions with the presented evidence, the authors should:

1. Reframe the study explicitly as a computational prioritization and biophysical characterization of candidate AMPs, rather than as a demonstration of antimicrobial mechanisms.

2 Replace mechanistic terminology (e.g., “dual mechanism,” “pore-forming,” “disruptive action”) with language describing interaction modes, orientation states, or membrane perturbation patterns inferred from simulations.

3. Clearly state the limitations of single-peptide MD simulations and avoid implying bactericidal function or therapeutic efficacy in the absence of experimental validation.

4. Ensure consistency of this revised framing across the title, abstract, Results, Discussion, and Conclusions.

5. Addressing this conceptual issue does not require additional experimental data, but it does require a careful and systematic revision of the manuscript’s interpretative framework.

6. The membrane models used for A. baumannii, K. pneumoniae, and P. aeruginosa are not equivalent. While A. baumannii is modeled using an asymmetric outer membrane containing Lipid A/LPS, K. pneumoniae and P. aeruginosa are represented by symmetric phospholipid bilayers lacking LPS. This distinction is not clearly justified or discussed, yet results are directly compared across systems. Given the known impact of LPS on peptide binding, insertion, and membrane perturbation, the authors should explicitly clarify the rationale for using different membrane architectures and discuss the implications and limitations this imposes on cross-species comparisons.

7. The in silico toxicity and hemolysis predictors suggest a favorable safety profile; the authors could include this in silico profile.

**********

6. PLOS authors have the option to publish the peer review history of their article (what does this mean?). If published, this will include your full peer review and any attached files.

If you choose “no”, your identity will remain anonymous but your review may still be made public.

**Do you want your identity to be public for this peer review?**  For information about this choice, including consent withdrawal, please see our Privacy Policy.

Reviewer #1: No

Reviewer #2: No

Reviewer #3: No

**********

[NOTE: If reviewer comments were submitted as an attachment file, they will be attached to this email and accessible via the submission site. Please log into your account, locate the manuscript record, and check for the action link "View Attachments". If this link does not appear, there are no attachment files.]

To ensure your figures meet our technical requirements, please review our figure guidelines: https://journals.plos.org/plosone/s/figures

You may also use PLOS’s free figure tool, NAAS, to help you prepare publication quality figures: https://journals.plos.org/plosone/s/figures#loc-tools-for-figure-preparation.

NAAS will assess whether your figures meet our technical requirements by comparing each figure against our figure specifications.

# Author comment: Author response to Decision Letter 1

**DOI:** 10.1371/journal.pone.0353985.r002

30 Mar 2026

Dear Editor,

We would like to express our sincere gratitude for acknowledging our manuscript and providing us the opportunity to revise it for possible publication in your esteemed journal of PLOS One.

We thank all the peer reviewers for their constructive and insightful suggestions towards our manuscript. Please find our detailed, point-by-point responses (in blue italics) to the reviewers’ comments below. We have carefully addressed each comment, conducted additional analyses where feasible, and amended the revised manuscript accordingly. We hope that our revised manuscript is now suitable for publication in the journal of “PLOS One”.

Point-by-point response reviewers’ comments

Editor

Comment:

1. The manuscript in its current form overstates the experimental implications of what is fundamentally a computational study. In the current form, manuscript overstates the functional activity of the peptides; all claims should be moderated. The title and several statements in the abstract, results, and discussion should be revised to explicitly indicate that this is an in silico investigation and that the reported antimicrobial activities are predicted rather than demonstrated.

Author’s Response

We sincerely thank the Editor for this important observation. We fully agree that the original manuscript language inadvertently implied experimental demonstration of antimicrobial activity. Accordingly, we have revised the title, abstract, results, and discussion to explicitly reflect the computational and predictive nature of this study. The revised title now reads: "In silico identification and biophysical characterization of candidate antimicrobial peptides from the Indian marine microbiome targeting multidrug-resistant ESKAPE pathogens." Throughout the manuscript, terms such as "dual mechanisms of disruptive action" and "functional peptide candidates" have been replaced with "distinct membrane interaction modes" and "candidate peptides," respectively, to ensure all claims are appropriately moderated and consistent with the in silico scope of the work. These changes have been made in the Title (Page 1), Abstract (Page 2), and Conclusion section (Section 5, Page 24) of the revised manuscript.

Comment:

2. The absence of any wet-lab validation is a significant limitation and either wet-lab experiments should be done or more clearly acknowledged, accompanied by a concrete experimental roadmap (e.g., MIC testing, membrane leakage, and cytotoxicity assays).

Author’s Response

We thank the Editor for raising this important point. We acknowledge that the absence of experimental validation is a significant limitation of the present study. As this work is a purely computational investigation, wet-lab experiments were beyond the scope of the current manuscript. However, we have now explicitly acknowledged this limitation and included a concrete experimental roadmap for future validation at the end of the Discussion section (Section 4, Page 22) of the revised manuscript. The proposed roadmap includes: (i) minimum inhibitory concentration (MIC) assays against representative ESKAPE strains, (ii) membrane permeabilization assays using fluorescent dye uptake (e.g., SYTOX Green or propidium iodide), (iii) membrane leakage assays using liposome models, and (iv) hemolysis and cytotoxicity assays on mammalian cell lines to assess selectivity and safety.

Comment:

3. The rationale for selecting only two peptides for molecular dynamics simulations is insufficiently justified and should either be clarified or expanded to include additional candidates.

Author’s Response

We thank the Editor for this important observation. We acknowledge that the rationale for selecting c_AMP_1 and c_AMP_2 for molecular dynamics simulations was not sufficiently articulated in the original manuscript. The manuscript already describes the upstream filtering steps, including a length cut-off of ≤50 amino acids, presence of arginine and tryptophan residues, and pLM4CPPs-based cell-penetrating peptide prediction, that reduced the candidate pool to 10 peptides (Section 3.1, Page 12). From these 10, c_AMP_1 and c_AMP_2 were specifically chosen for all-atom MD simulations based on a multi-criteria evaluation: (i) highest consensus scores across all six machine learning prediction tools, (ii) contrasting yet complementary physicochemical profiles - c_AMP_1 exhibiting a high net positive charge (+11) and strong amphiphilicity (1.59), while c_AMP_2 showed moderate charge (+6) and balanced hydrophobicity (0.89), (iii) structurally distinct AlphaFold3-predicted conformations, c_AMP_1 adopting a continuous α-helical structure and c_AMP_2 a semi-helical conformation with flexible termini, enabling a meaningful comparative biophysical analysis, and (iv) the considerable computational demands of 300 ns all-atom simulations across three bacterial membrane systems, which necessitated a focused selection of the most structurally divergent candidates. This rationale has now been explicitly clarified in Results (Section 3.2, Page 13) and Methods (Section 2.4, Page 8) of the revised manuscript.

Comment:

4. Furthermore, given the reliance on Arg/Trp-based rules for cell-penetrating behavior, the authors should cross-validate their shortlisted peptides using established CPP resources such as CPPsite 2.0, CellPPDmod.

Author’s Response

We thank the Editor for this valuable suggestion. As recommended, we have cross-validated all ten shortlisted peptides using CellPPD-Mod and CPPsite 3.0 (an updated version of CPPsite 2.0), in addition to the originally used pLM4CPPs model. CPP activity was independently predicted using two machine learning-based tools - pLM4CPPs and CellPPD-Mod. Nine out of ten peptides were consistently predicted as CPPs by both tools. One peptide, c_AMP_6, yielded conflicting predictions, classified as CPP by pLM4CPPs but as Non-CPP by CellPPD-Mod, and this discrepancy has been duly acknowledged in the revised manuscript. Additionally, similarity searches against the CPPsite 3.0 database of experimentally validated CPPs using the Smith-Waterman algorithm were conducted as a complementary novelty assessment. All ten candidates yielded low alignment scores (13-19) over short local windows (3-9 residues), indicating no significant full-length similarity to any known CPP in the database. The prediction thresholds applied for each tool have been clearly stated in the Methods section. These results have been incorporated into Methods (Section 2.4, Page 8) and Results (Section 3.1, Page 12) of the revised manuscript and summarized in Supplementary Tables S3 and S4.

Comment:

5. In addition, many experimentally validated antitubercular peptides are absent from general AMP repositories used to train current predictors, which can affect sensitivity for Mycobacterium-active peptides. Therefore, benchmarking against specialized datasets and tools such as AntiTbPdb and AntiTbPred would also help assess the sensitivity of their pipeline, particularly for anti-mycobacterial relevance.

Author’s Response

We thank the Editor for this insightful comment. We acknowledge that general AMP prediction tools may underrepresent experimentally validated antitubercular peptides in their training datasets, potentially limiting the sensitivity of the pipeline for identifying Mycobacterium-active peptides. We wish to clarify that the primary scope of the present study is the identification of candidate AMPs targeting Gram-negative ESKAPE pathogens, specifically Pseudomonas aeruginosa, Acinetobacter baumannii, and Klebsiella pneumoniae, and not Mycobacterium tuberculosis. Nevertheless, in response to the Editor's suggestion, we have conducted an exploratory screening of all ten shortlisted candidate peptides using AntiTbPred. All ten candidates were predicted as potential anti-tubercular peptides, with scores ranging from 0.26 to 1.72, suggesting possible broad-spectrum activity that warrants further investigation. We have clearly stated in the revised manuscript that this analysis is purely exploratory in nature and that any anti-mycobacterial activity requires dedicated experimental validation using specialized assays. A note acknowledging the limitation of general AMP predictors for Mycobacterium-specific activity and recommending AntiTbPdb-based benchmarking as a direction for future work has also been added. These additions have been incorporated into the Discussion section (Section 4, Page 23) of the revised manuscript. The AntiTbPred results are summarized in Supplementary Table S5.

Comment:

6. Finally, the translational framing would be strengthened by comparing the lead peptides with characteristics of clinically approved therapeutics using curated resources such as THPdb, SATPdb etc. and by more deeply engaging with recent marine AMP and membrane-modeling literature.

Author’s Response

We thank the Editor for this constructive suggestion. In response to the first part of this comment, similarity searches were performed for c_AMP_1 and c_AMP_2 against THPdb2, a curated database of FDA-approved therapeutic peptides and proteins, using the Smith-Waterman algorithm. No statistically significant matches were identified, c_AMP_1 returned a best hit with an E-value of 1.7 and c_AMP_2 with an E-value of 6.2, confirming that both lead peptides are structurally distinct from currently approved therapeutic peptides. Importantly, both candidates fall within the "Long Peptides (21-50 AA)" category of THPdb2, which encompasses 45 unique FDA-approved therapeutic peptides, indicating that their length profile is consistent with clinically approved peptide therapeutics. Furthermore, their strongly cationic nature, amphipathic architecture, and predicted membrane-targeting properties align well with the physicochemical characteristics associated with approved antimicrobial peptide therapeutics, collectively supporting their translational potential as scaffolds for future peptide-based drug development. These observations have been incorporated into the Discussion section (Section 4, Page 23) of the revised manuscript, and the THPdb2 similarity search results are provided in Supplementary Table S6. In response to the second part of this comment, we have expanded our engagement with recent marine AMP discovery and membrane-modeling literature by incorporating additional relevant citations in the Introduction and Discussion sections of the revised manuscript. All newly added references are highlighted in yellow in the reference section of the revised manuscript for the Editor's and Reviewer's convenience.

Reviewer #1

Comments:

1. Dear Author, the article titled "Uncovering dual-mechanism antimicrobial peptides from the Indian marine microbiome to combat multidrug-resistant ESKAPE Pathogens" was reviewed. This article provides useful information for its readers. Please make the following correction:

Some references are outdated, please use the new references.

Authors' Response

We thank the Reviewer for this comment. We have carefully reviewed all citations in the manuscript and updated or supplemented references where more recent publications are available. References pertaining to specific software tools and prediction algorithms - including MEGAHIT (Li et al., 2016), CHARMM-GUI (Lee et al., 2016), FATSLiM (Buchoux, 2017), CONAN (Mercadante et al., 2018), AMPScanner v2 (Veltri et al., 2018), and APIN (Su et al., 2019) - have been retained as their original publications, as these are the primary citations for the respective tools. For conceptual and mechanistic citations, the following older references have been supplemented with more recent literature: (i) Khandelia & Kaznessis (2007) supplemented with Necula et al. (2023) for cation-π interactions in AMP membrane binding; (ii) Kooijman et al. (2007) supplemented with Graber et al. (2022) for hydrogen bond interactions with phospholipid headgroups; (iii) Mukherjee et al. (2017) supplemented with Ma et al. (2024) for membrane thinning in MD simulations; (iv) Hong et al. (2019) supplemented with Sun et al. (2022) for melittin membrane insertion; and (v) Bobone & Stella (2019) replaced with Ma et al. (2024) for AMP selectivity toward bacterial membranes. All newly added references are highlighted in yellow in the reference section of the revised manuscript for the Editor's and Reviewer's convenience. These updates have been incorporated throughout the revised manuscript.

Reviewer #2

The authors analyze five Indian marine metagenomic datasets to identify antimicrobial peptide candidates using six prediction tools. After applying additional filters based on peptide length, amino acid composition, and predicted cell-penetrating properties, they reduce the set to ten peptides. Two of these are then selected for molecular dynamics simulations with Gram-negative membrane models. The differences in how these peptides interact with the membranes are described in the Results and discussed as possible dual mechanisms. While the overall workflow is logical and clearly presented, several aspects of the methodology and interpretation require clarification and more cautious framing.

Author’s Response

We sincerely thank the Reviewer for this thoughtful summary of our work and for acknowledging the logical and clear presentation of the overall workflow. We appreciate the constructive feedback regarding the areas requiring clarification and more cautious framing. We fully agree with the Reviewer's assessment that several aspects of the methodology and interpretation required additional clarity and moderation, particularly with respect to the characterization of the observed peptide-membrane interaction differences as "dual mechanisms." We have carefully addressed each of the specific concerns raised, as detailed in our point-by-point responses below. The manuscript has been revised accordingly to provide greater methodological transparency and to ensure that all interpretations are appropriately aligned with the in silico scope of the study.

Comment:

1. The Methods section should clearly state (i) the total number of peptides advanced to MD simulations, (ii)Whether c_AMP_1 and c_AMP_2 were the only peptides simulated, and (iii) the criteria used to select them (Charge, hydrophobic moment, predictable activity, or structural diversity).

Author’s Response

We thank the Reviewer for this important observation. We confirm that c_AMP_1 and c_AMP_2 were the only two peptides advanced to all-atom MD simulations in this study. Their selection from the ten shortlisted candidates was based on a multi-criteria evaluation framework considering: (i) net charge - c_AMP_1 exhibiting the highest net positive charge (+11) and c_AMP_2 a moderate charge (+6), representing contrasting electrostatic profiles; (ii) hydrophobic moment and amphiphilicity index - c_AMP_1 with 1.59 and c_AMP_2 with 0.89, reflecting distinct hydrophobic distributions; (iii) predicted membrane activity based on physicochemical profiling via DBAASP; and (iv) structural diversity - c_AMP_1 adopting a continuous α-helical conformation and c_AMP_2 a semi-helical structure with flexible termini as predicted by AlphaFold3. These clarifications have now been explicitly incorporated into Methods (Section 2.4, Page 8) and Results (Section 3.2, Page 13) of the revised manuscript.

Comment:

2. The claim of "dual mechanisms" seems somewhat stronger than what is directly supported by the simulation results. The observed differences mainly show distinct membrane interaction behaviors in the MD simulations, rather than clearly separate antimicrobial mechanisms. The reported analyses describe interaction patterns but do not directly demonstrate effects such as pore formation or membrane permeabilization. The authors may consider softening the wording in the Discussion and Conclusions to better reflect the in silico scope of the results.

Author’s Response

We thank the Reviewer for this important and valid observation. We fully agree that the MD simulation data describe distinct membrane interaction modes and orientation behaviors rather than directly demonstrating functional antimicrobial mechanisms such as pore formation or membrane permeabilization. Accordingly, we have carefully revised the Discussion and Conclusions sections

Attachment Submitted filename: Response to Reviewers.docx

# Peer review: Decision Letter 1

**DOI:** 10.1371/journal.pone.0353985.r003

**Authors:** Salman Sadullah Usmani, Salman Sadullah Usmani

14 Apr 2026

PONE-D-25-55465R1

In silico identification and biophysical characterization of candidate antimicrobial peptides from the Indian marine microbiome targeting multidrug-resistant ESKAPE pathogens

PLOS One

Dear Dr. Dehury,

Thank you for submitting your manuscript to PLOS ONE. After careful consideration, we feel that it has merit but does not fully meet PLOS ONE’s publication criteria as it currently stands. Therefore, we invite you to submit a revised version of the manuscript that addresses the points raised during the review process.

Please submit your revised manuscript by May 29 2026 11:59PM. If you will need more time than this to complete your revisions, please reply to this message or contact the journal office at plosone@plos.org. When you're ready to submit your revision, log on to https://www.editorialmanager.com/pone/ and select the 'Submissions Needing Revision' folder to locate your manuscript file.

Please include the following items when submitting your revised manuscript:

- A letter that responds to each point raised by the academic editor and reviewer(s). You should upload this letter as a separate file labeled 'Response to Reviewers'.
- A marked-up copy of your manuscript that highlights changes made to the original version. You should upload this as a separate file labeled 'Revised Manuscript with Track Changes'.
- An unmarked version of your revised paper without tracked changes. You should upload this as a separate file labeled 'Manuscript'.

If you would like to make changes to your financial disclosure, please include your updated statement in your cover letter. Guidelines for resubmitting your figure files are available below the reviewer comments at the end of this letter.

If applicable, we recommend that you deposit your laboratory protocols in protocols.io to enhance the reproducibility of your results. Protocols.io assigns your protocol its own identifier (DOI) so that it can be cited independently in the future. For instructions see: https://journals.plos.org/plosone/s/submission-guidelines#loc-laboratory-protocols. Additionally, PLOS ONE offers an option for publishing peer-reviewed Lab Protocol articles, which describe protocols hosted on protocols.io. Read more information on sharing protocols at https://plos.org/protocols?utm_medium=editorial-email&utm_source=authorletters&utm_campaign=protocols.

As the corresponding author, your ORCID iD is verified in the submission system and will appear in the published article. PLOS supports the use of ORCID, and we encourage all coauthors to register for an ORCID iD and use it as well. Please encourage your coauthors to verify their ORCID iD within the submission system before final acceptance, as unverified ORCID iDs will not appear in the published article. *Only*  the individual author can complete the verification step; PLOS staff *cannot*  verify ORCID iDs on behalf of authors.

We look forward to receiving your revised manuscript.

Kind regards,

Salman Sadullah Usmani, Ph.D.

Academic Editor

PLOS One

Journal Requirements:

If the reviewer comments include a recommendation to cite specific previously published works, please review and evaluate these publications to determine whether they are relevant and should be cited. There is no requirement to cite these works unless the editor has indicated otherwise.

Please review your reference list to ensure that it is complete and correct. If you have cited papers that have been retracted, please include the rationale for doing so in the manuscript text, or remove these references and replace them with relevant current references. Any changes to the reference list should be mentioned in the rebuttal letter that accompanies your revised manuscript. If you need to cite a retracted article, indicate the article’s retracted status in the References list and also include a citation and full reference for the retraction notice.

[Note: HTML markup is below. Please do not edit.]

Reviewers' comments:

Reviewer's Responses to Questions

**Comments to the Author**

1. If the authors have adequately addressed your comments raised in a previous round of review and you feel that this manuscript is now acceptable for publication, you may indicate that here to bypass the “Comments to the Author” section, enter your conflict of interest statement in the “Confidential to Editor” section, and submit your "Accept" recommendation.

Reviewer #2: All comments have been addressed

Reviewer #3: All comments have been addressed

**********

2. Is the manuscript technically sound, and do the data support the conclusions?

The manuscript must describe a technically sound piece of scientific research with data that supports the conclusions. Experiments must have been conducted rigorously, with appropriate controls, replication, and sample sizes. The conclusions must be drawn appropriately based on the data presented.

Reviewer #2: Partly

Reviewer #3: Yes

**********

3. Has the statistical analysis been performed appropriately and rigorously?

Reviewer #2: N/A

Reviewer #3: N/A

**********

4. Have the authors made all data underlying the findings in their manuscript fully available?

The PLOS Data policy requires authors to make all data underlying the findings described in their manuscript fully available without restriction, with rare exception (please refer to the Data Availability Statement in the manuscript PDF file). The data should be provided as part of the manuscript or its supporting information, or deposited to a public repository. For example, in addition to summary statistics, the data points behind means, medians and variance measures should be available. If there are restrictions on publicly sharing data—e.g. participant privacy or use of data from a third party—those must be specified.

Reviewer #2: Yes

Reviewer #3: Yes

**********

5. Is the manuscript presented in an intelligible fashion and written in standard English?

PLOS ONE does not copyedit accepted manuscripts, so the language in submitted articles must be clear, correct, and unambiguous. Any typographical or grammatical errors should be corrected at revision, so please note any specific errors here.

Reviewer #2: Yes

Reviewer #3: Yes

**********

6. Review Comments to the Author

Please use the space provided to explain your answers to the questions above. You may also include additional comments for the author, including concerns about dual publication, research ethics, or publication ethics. (Please upload your review as an attachment if it exceeds 20,000 characters)

Reviewer #2: The authors have satisfactorily addressed the concerns raised in the previous round of review. The manuscript has improved in terms of clarity, methodological description, and overall framing.

In particular, the interpretation of the molecular dynamics results has been revised appropriately, and the conclusions are now better aligned with the in-silico nature of the study. The rationale for peptide selection and the description of the simulation setup is now clearer. The manuscript also includes a more explicit discussion of its limitations, including the absence of experimental validation and independent benchmarking.

One minor point remains regarding consistency of wording. In a few sections, particularly the conclusion, the language still slightly overemphasizes potential therapeutic implications. This should be moderated to ensure it remains fully consistent with the computational scope of the study.

Overall, the manuscript is now scientifically sound and suitable for publication after minor revision.

Reviewer #3: (No Response)

**********

7. PLOS authors have the option to publish the peer review history of their article (what does this mean?). If published, this will include your full peer review and any attached files.

If you choose “no”, your identity will remain anonymous but your review may still be made public.

**Do you want your identity to be public for this peer review?**  For information about this choice, including consent withdrawal, please see our Privacy Policy.

Reviewer #2: No

Reviewer #3: No

**********

[NOTE: If reviewer comments were submitted as an attachment file, they will be attached to this email and accessible via the submission site. Please log into your account, locate the manuscript record, and check for the action link "View Attachments". If this link does not appear, there are no attachment files.]

To ensure your figures meet our technical requirements, please review our figure guidelines: https://journals.plos.org/plosone/s/figures

You may also use PLOS’s free figure tool, NAAS, to help you prepare publication quality figures: https://journals.plos.org/plosone/s/figures#loc-tools-for-figure-preparation.

NAAS will assess whether your figures meet our technical requirements by comparing each figure against our figure specifications.

# Author comment: Author response to Decision Letter 2

**DOI:** 10.1371/journal.pone.0353985.r004

16 Apr 2026

Dear Editor,

We would like to express our sincere gratitude for acknowledging our manuscript and providing us the opportunity to revise it for possible publication in your esteemed journal of PLOS One.

We thank all the peer reviewers for their constructive and insightful suggestions towards our manuscript. Please find our detailed, point-by-point responses (in blue italics) to the reviewers’ comments below. We have carefully addressed each comment, conducted additional analyses where feasible, and amended the revised manuscript accordingly. We hope that our revised manuscript is now suitable for publication in the journal of “PLOS One”.

Point-by-point response reviewers’ comments

Reviewer #2

The authors have satisfactorily addressed the concerns raised in the previous round of review. The manuscript has improved in terms of clarity, methodological description, and overall framing. In particular, the interpretation of the molecular dynamics results has been revised appropriately, and the conclusions are now better aligned with the in-silico nature of the study. The rationale for peptide selection and the description of the simulation setup is now clearer. The manuscript also includes a more explicit discussion of its limitations, including the absence of experimental validation and independent benchmarking.

Author’s Response

We sincerely thank the Reviewer for this positive and encouraging assessment of the revised manuscript. We are glad that the revisions addressing the interpretation of molecular dynamics results, the clarity of the peptide selection rationale, the simulation setup description, and the explicit discussion of limitations have met the Reviewer's expectations. We have further addressed the single remaining minor point identified below.

Comment:

1. One minor point remains regarding consistency of wording. In a few sections, particularly the conclusion, the language still slightly overemphasizes potential therapeutic implications. This should be moderated to ensure it remains fully consistent with the computational scope of the study.

Author’s Response

We thank the Reviewer for this observation. We agree that the language in a few sections inadvertently overemphasized the therapeutic implications of the study beyond what is directly supported by the in silico evidence. Accordingly, we have carefully reviewed the manuscript and made targeted revisions in the Abstract (Page 2), Introduction (Section 1, Page 6), Discussion (Section 4, Page 23) and Conclusion (Section 5, Page 24) to moderate such language. Phrases implying confirmed findings or direct therapeutic applicability have been replaced with appropriately qualified expressions that accurately reflect the computational and predictive scope of the study. We believe these revisions ensure that the manuscript is now fully consistent in its framing throughout.

Reviewer #3

No comments were provided.

Author’s Response

We thank the reviewer for recommending our work for publication.

Attachment Submitted filename: Response_to_Reviewers_auresp_2.docx

# Peer review: Decision Letter 2

**DOI:** 10.1371/journal.pone.0353985.r005

**Authors:** Salman Sadullah Usmani, Salman Sadullah Usmani, Salman Sadullah Usmani

15 Jun 2026

PONE-D-25-55465R2

In silico identification and biophysical characterization of candidate antimicrobial peptides from the Indian marine microbiome targeting multidrug-resistant ESKAPE pathogens

PLOS One

Dear Dr. Dehury,

Thank you for submitting your manuscript to PLOS ONE. After careful consideration, we feel that it has merit but does not fully meet PLOS ONE’s publication criteria as it currently stands. Therefore, we invite you to submit a revised version of the manuscript that addresses the points raised during the review process.

Please submit your revised manuscript by Jul 30 2026 11:59PM. If you will need more time than this to complete your revisions, please reply to this message or contact the journal office at plosone@plos.org. When you're ready to submit your revision, log on to https://www.editorialmanager.com/pone/ and select the 'Submissions Needing Revision' folder to locate your manuscript file.

Please include the following items when submitting your revised manuscript:

- A letter that responds to each point raised by the academic editor and reviewer(s). You should upload this letter as a separate file labeled 'Response to Reviewers'.
- A marked-up copy of your manuscript that highlights changes made to the original version. You should upload this as a separate file labeled 'Revised Manuscript with Track Changes'.
- An unmarked version of your revised paper without tracked changes. You should upload this as a separate file labeled 'Manuscript'.

If you would like to make changes to your financial disclosure, please include your updated statement in your cover letter. Guidelines for resubmitting your figure files are available below the reviewer comments at the end of this letter.

If applicable, we recommend that you deposit your laboratory protocols in protocols.io to enhance the reproducibility of your results. Protocols.io assigns your protocol its own identifier (DOI) so that it can be cited independently in the future. For instructions see: https://journals.plos.org/plosone/s/submission-guidelines#loc-laboratory-protocols. Additionally, PLOS ONE offers an option for publishing peer-reviewed Lab Protocol articles, which describe protocols hosted on protocols.io. Read more information on sharing protocols at https://plos.org/protocols?utm_medium=editorial-email&utm_source=authorletters&utm_campaign=protocols.

As the corresponding author, your ORCID iD is verified in the submission system and will appear in the published article. PLOS supports the use of ORCID, and we encourage all coauthors to register for an ORCID iD and use it as well. Please encourage your coauthors to verify their ORCID iD within the submission system before final acceptance, as unverified ORCID iDs will not appear in the published article. *Only*  the individual author can complete the verification step; PLOS staff *cannot*  verify ORCID iDs on behalf of authors.

We look forward to receiving your revised manuscript.

Kind regards,

Salman Sadullah Usmani, Ph.D.

Academic Editor

PLOS One

Journal Requirements:

If the reviewer comments include a recommendation to cite specific previously published works, please review and evaluate these publications to determine whether they are relevant and should be cited. There is no requirement to cite these works unless the editor has indicated otherwise.

Please review your reference list to ensure that it is complete and correct. If you have cited papers that have been retracted, please include the rationale for doing so in the manuscript text, or remove these references and replace them with relevant current references. Any changes to the reference list should be mentioned in the rebuttal letter that accompanies your revised manuscript. If you need to cite a retracted article, indicate the article’s retracted status in the References list and also include a citation and full reference for the retraction notice.

[Note: HTML markup is below. Please do not edit.]

Reviewers' comments:

Reviewer's Responses to Questions

**Comments to the Author**

1. If the authors have adequately addressed your comments raised in a previous round of review and you feel that this manuscript is now acceptable for publication, you may indicate that here to bypass the “Comments to the Author” section, enter your conflict of interest statement in the “Confidential to Editor” section, and submit your "Accept" recommendation.

Reviewer #2: All comments have been addressed

**********

2. Is the manuscript technically sound, and do the data support the conclusions?

The manuscript must describe a technically sound piece of scientific research with data that supports the conclusions. Experiments must have been conducted rigorously, with appropriate controls, replication, and sample sizes. The conclusions must be drawn appropriately based on the data presented.

Reviewer #2: Partly

**********

3. Has the statistical analysis been performed appropriately and rigorously?

Reviewer #2: N/A

**********

4. Have the authors made all data underlying the findings in their manuscript fully available?

The PLOS Data policy requires authors to make all data underlying the findings described in their manuscript fully available without restriction, with rare exception (please refer to the Data Availability Statement in the manuscript PDF file). The data should be provided as part of the manuscript or its supporting information, or deposited to a public repository. For example, in addition to summary statistics, the data points behind means, medians and variance measures should be available. If there are restrictions on publicly sharing data—e.g. participant privacy or use of data from a third party—those must be specified.

Reviewer #2: No

**********

5. Is the manuscript presented in an intelligible fashion and written in standard English?

PLOS ONE does not copyedit accepted manuscripts, so the language in submitted articles must be clear, correct, and unambiguous. Any typographical or grammatical errors should be corrected at revision, so please note any specific errors here.

Reviewer #2: Yes

**********

6. Review Comments to the Author

Please use the space provided to explain your answers to the questions above. You may also include additional comments for the author, including concerns about dual publication, research ethics, or publication ethics. (Please upload your review as an attachment if it exceeds 20,000 characters)

Reviewer #2: The revised manuscript is improved compared to the previous version, and the authors have addressed many of the earlier concerns. The topic is important, and the overall workflow of the

study is presented clearly.

Some parts of the manuscript still sound stronger than what is shown in the study. The MD simulations show different ways the peptides interact with the membranes, but they do not directly

prove antimicrobial mechanisms or therapeutic effects.

The wording in the Abstract, Results, Discussion and Conclusion should be made more careful and consistent with the computational nature of the work.

There are still a few inconsistencies regarding the peptides used for the MD simulations. In some sections, three peptides are mentioned, while the detailed analysis is only presented for c_AMP_1

and c_AMP_2. This should be corrected throughout the manuscript.

In some places, the interpretation of the membrane interaction results goes beyond what can be directly concluded from the simulations. Since no experimental validation has been included, these points should be discussed more cautiously.

Overall, the manuscript has improved after revision, but some additional corrections are still needed to improve clarity and maintain consistency throughout the study

**********

7. PLOS authors have the option to publish the peer review history of their article (what does this mean?). If published, this will include your full peer review and any attached files.

If you choose “no”, your identity will remain anonymous but your review may still be made public.

**Do you want your identity to be public for this peer review?**  For information about this choice, including consent withdrawal, please see our Privacy Policy.

Reviewer #2: No

**********

[NOTE: If reviewer comments were submitted as an attachment file, they will be attached to this email and accessible via the submission site. Please log into your account, locate the manuscript record, and check for the action link "View Attachments". If this link does not appear, there are no attachment files.]

To ensure your figures meet our technical requirements, please review our figure guidelines: https://journals.plos.org/plosone/s/figures

You may also use PLOS’s free figure tool, NAAS, to help you prepare publication quality figures: https://journals.plos.org/plosone/s/figures#loc-tools-for-figure-preparation.

NAAS will assess whether your figures meet our technical requirements by comparing each figure against our figure specifications.

# Author comment: Author response to Decision Letter 3

**DOI:** 10.1371/journal.pone.0353985.r006

22 Jun 2026

Dear Editor,

We would like to express our sincere gratitude for acknowledging our manuscript and providing us the opportunity to revise it for possible publication in your esteemed journal of PLOS One.

We thank all the peer reviewers for their constructive and insightful suggestions towards our manuscript. Please find our detailed, point-by-point responses (in blue italics) to the reviewer’s comments below. We have carefully addressed each comment and amended the revised manuscript accordingly, with all changes marked using track changes. We hope that our revised manuscript is now suitable for publication in the journal of “PLOS One”.

Point-by-point response reviewers’ comments

Reviewer #2

The revised manuscript is improved compared to the previous version, and the authors have addressed many of the earlier concerns. The topic is important, and the overall workflow of the

study is presented clearly.

Author’s Response

We sincerely thank the Reviewer for the careful re-evaluation of our manuscript and for acknowledging the improvements made in the revised version. We are grateful for the recognition that the topic is important and that the overall workflow is presented clearly. We have taken the remaining comments seriously and have made targeted revisions throughout the Abstract, Results, Discussion, and Conclusion to ensure that the language is fully consistent with the computational and predictive nature of the study, that the membrane-interaction results are interpreted cautiously in the absence of experimental validation, and that the number of peptides used for the molecular dynamics simulations is stated unambiguously. Our point-by-point responses are provided below, and all corresponding changes have been marked using track changes in the revised manuscript.

Comment:

1. Some parts of the manuscript still sound stronger than what is shown in the study. The MD simulations show different ways the peptides interact with the membranes, but they do not directly prove antimicrobial mechanisms or therapeutic effects.

Author’s Response

We thank the Reviewer for this important observation. We fully agree that the molecular dynamics simulations characterize predicted peptide-membrane interaction modes and do not, in themselves, demonstrate antimicrobial mechanisms or therapeutic effects. Accordingly, we have revised the concluding statements of the Abstract (Page 2), the Discussion (Section 4, Page 23), and the Conclusion (Section 5, Page 24) to remove wording that could imply proven activity or therapeutic readiness. Expressions such as “effectively prioritizes,” “conducive to therapeutic development,” and “strongly support” have been replaced with appropriately qualified phrasing that frames the peptides as computational candidates requiring experimental validation. We note that the manuscript already contains a dedicated paragraph in the Discussion explicitly stating that single-peptide MD simulations cannot establish pore formation, permeabilization, or bactericidal activity; the present revisions bring the surrounding summary statements into line with that caveat.

Comment:

2. The wording in the Abstract, Results, Discussion and Conclusion should be made more careful and consistent with the computational nature of the work.

Author’s Response

We thank the Reviewer for this suggestion and have carried out a careful pass across the Abstract, Results, Discussion, and Conclusion to harmonize the language with the computational scope of the work. Subjective or comparative terms that implied functional superiority, such as “underperformed” and “superior membrane adaptability” in Results (Section 3.5, Page 17), have been rephrased in neutral, observation-based terms tied explicitly to the simulated systems. Verbs describing simulation outcomes such as “revealed,” “exhibited” have, where appropriate, been softened to “indicated” or “displayed,” and qualifiers such as “in the simulations” / “in silico” have been retained or added so that descriptive and interpretive statements read consistently throughout. Together with the changes made under the preceding and following comments, these edits ensure a uniform, computationally qualified tone across all four sections.

Comment:

3. There are still a few inconsistencies regarding the peptides used for the MD simulations. In some sections, three peptides are mentioned, while the detailed analysis is only presented for c_AMP_1 and c_AMP_2. This should be corrected throughout the manuscript.

Author’s Response

We thank the Reviewer for highlighting this point and apologize for the lack of clarity. We confirm that exactly two peptides, c_AMP_1 and c_AMP_2, were advanced to molecular dynamics simulations; all detailed analyses correspond to these two peptides. The recurring reference to “three” in the manuscript denotes the three bacterial membrane systems (A. baumannii, K. pneumoniae, and P. aeruginosa), not three peptides, and the close juxtaposition of “two peptides” and “three membranes” may have caused this ambiguity. To remove any possible confusion, we have revised the simulation-design statement in Materials and methods (Section 2.5, Page 9) to state the design explicitly as “two peptides × three membranes = six independent MD systems,” and we have re-checked the Abstract, Methods (Sections 2.4-2.5), Results (Sections 3.3-3.6), and Discussion (Section 4) to ensure that the two-peptide selection and the three-membrane design are stated consistently and cannot be conflated. The peptide count is now unambiguous throughout.

Comment:

4. In some places, the interpretation of the membrane interaction results goes beyond what can be directly concluded from the simulations. Since no experimental validation has been included, these points should be discussed more cautiously.

Author’s Response

We agree with the Reviewer that, in the absence of experimental validation, certain interpretations were stated more definitively than the simulations permit. We have therefore revised these statements to distinguish what is directly observed in the trajectories from what would require experimental confirmation. In Results (Section 3.5, Page 17), the causal phrasing “indicating that c_AMP_2 inserts more deeply… and induces localized lipid compression” has been changed to the observation-level “consistent with deeper insertion… within the simulated bilayers.” In the Discussion (Section 4, Page 21), the inference that a deeper orientation “could enhance permeabilization” has been qualified to state explicitly that permeabilization cannot be inferred from structural simulations alone and requires dedicated experimental assays. These revisions complement the existing limitations paragraph in the Discussion, which already notes that single-peptide simulations cannot establish pore formation or bactericidal activity, and ensure that all mechanistic interpretations are now framed as predicted, simulation-derived observations.

Attachment Submitted filename: Response_to_Reviewers_auresp_3.docx

# Peer review: Decision Letter 3

**DOI:** 10.1371/journal.pone.0353985.r007

**Authors:** Salman Sadullah Usmani, Salman Sadullah Usmani, Salman Sadullah Usmani, Salman Sadullah Usmani

2 Jul 2026

In silico identification and biophysical characterization of candidate antimicrobial peptides from the Indian marine microbiome targeting multidrug-resistant ESKAPE pathogens

PONE-D-25-55465R3

Dear Dr. Dehury,

We’re pleased to inform you that your manuscript has been judged scientifically suitable for publication and will be formally accepted for publication once it meets all outstanding technical requirements.

Within one week, you’ll receive an e-mail detailing the required amendments. When these have been addressed, you’ll receive a formal acceptance letter and your manuscript will be scheduled for publication.

An invoice will be generated when your article is formally accepted. Please note, if your institution has a publishing partnership with PLOS and your article meets the relevant criteria, all or part of your publication costs will be covered. Please make sure your user information is up-to-date by logging into Editorial Manager at Editorial Manager® and clicking the ‘Update My Information' link at the top of the page. For questions related to billing, please contact billing support.

If your institution or institutions have a press office, please notify them about your upcoming paper to help maximize its impact. If they’ll be preparing press materials, please inform our press team as soon as possible -- no later than 48 hours after receiving the formal acceptance. Your manuscript will remain under strict press embargo until 2 pm Eastern Time on the date of publication. For more information, please contact onepress@plos.org.

Kind regards,

Salman Sadullah Usmani, Ph.D.

Academic Editor

PLOS One

Additional Editor Comments (optional):

Reviewers' comments:

Reviewer's Responses to Questions

**Comments to the Author**

1. If the authors have adequately addressed your comments raised in a previous round of review and you feel that this manuscript is now acceptable for publication, you may indicate that here to bypass the “Comments to the Author” section, enter your conflict of interest statement in the “Confidential to Editor” section, and submit your "Accept" recommendation.

Reviewer #2: All comments have been addressed

**********

2. Is the manuscript technically sound, and do the data support the conclusions?

The manuscript must describe a technically sound piece of scientific research with data that supports the conclusions. Experiments must have been conducted rigorously, with appropriate controls, replication, and sample sizes. The conclusions must be drawn appropriately based on the data presented.

Reviewer #2: Yes

**********

3. Has the statistical analysis been performed appropriately and rigorously?

Reviewer #2: N/A

**********

4. Have the authors made all data underlying the findings in their manuscript fully available?

The PLOS Data policy requires authors to make all data underlying the findings described in their manuscript fully available without restriction, with rare exception (please refer to the Data Availability Statement in the manuscript PDF file). The data should be provided as part of the manuscript or its supporting information, or deposited to a public repository. For example, in addition to summary statistics, the data points behind means, medians and variance measures should be available. If there are restrictions on publicly sharing data—e.g. participant privacy or use of data from a third party—those must be specified.

Reviewer #2: Yes

**********

5. Is the manuscript presented in an intelligible fashion and written in standard English?

PLOS ONE does not copyedit accepted manuscripts, so the language in submitted articles must be clear, correct, and unambiguous. Any typographical or grammatical errors should be corrected at revision, so please note any specific errors here.

Reviewer #2: Yes

**********

6. Review Comments to the Author

Please use the space provided to explain your answers to the questions above. You may also include additional comments for the author, including concerns about dual publication, research ethics, or publication ethics. (Please upload your review as an attachment if it exceeds 20,000 characters)

Reviewer #2: The revised manuscript is improved, and the authors have addressed my previous comments satisfactorily. The interpretation is now more appropriately cautious and consistent with the computational nature of the study. I have o further concerns.

**********

7. PLOS authors have the option to publish the peer review history of their article (what does this mean?). If published, this will include your full peer review and any attached files.

If you choose “no”, your identity will remain anonymous but your review may still be made public.

**Do you want your identity to be public for this peer review?**  For information about this choice, including consent withdrawal, please see our Privacy Policy.

Reviewer #2: No

**********

# Editor report: Acceptance letter

**DOI:** 10.1371/journal.pone.0353985.r008

**Authors:** Salman Sadullah Usmani, Salman Sadullah Usmani, Salman Sadullah Usmani, Salman Sadullah Usmani

PONE-D-25-55465R3

PLOS One

Dear Dr. Dehury,

I'm pleased to inform you that your manuscript has been deemed suitable for publication in PLOS One. Congratulations! Your manuscript is now being handed over to our production team.

At this stage, our production department will prepare your paper for publication. This includes ensuring the following:

* All references, tables, and figures are properly cited

* All relevant supporting information is included in the manuscript submission,

* There are no issues that prevent the paper from being properly typeset

You will receive further instructions from the production team, including instructions on how to review your proof when it is ready. Please keep in mind that we are working through a large volume of accepted articles, so please give us a few days to review your paper and let you know the next and final steps.

Lastly, if your institution or institutions have a press office, please let them know about your upcoming paper now to help maximize its impact. If they'll be preparing press materials, please inform our press team within the next 48 hours. Your manuscript will remain under strict press embargo until 2 pm Eastern Time on the date of publication. For more information, please contact onepress@plos.org.

You will receive an invoice from PLOS for your publication fee after your manuscript has reached the completed accept phase. If you receive an email requesting payment before acceptance or for any other service, this may be a phishing scheme. Learn how to identify phishing emails and protect your accounts at https://explore.plos.org/phishing.

If we can help with anything else, please email us at customercare@plos.org.

Thank you for submitting your work to PLOS ONE and supporting open access.

Kind regards,

PLOS ONE Editorial Office Staff

on behalf of

Dr. Salman Sadullah Usmani

Academic Editor

PLOS One
